# Characterizing the supra- and subsolidus processes that generated the Current PGE–Cu–Ni deposit, Thunder Bay North Intrusive Complex, Canada: insights from trace elements and multiple S isotopes of sulfides

**DOI:** 10.1007/s00126-023-01193-9

**Published:** 2023-07-29

**Authors:** M. Brzozowski, P. Hollings, G. Heggie, A. MacTavish, D. Wilton, D. Evans-Lamswood

**Affiliations:** 1 British Columbia Geological Survey, 1810 Blanshard Street, Victoria, BC V8T 4J1 Canada; 2 Department of Geology, Lakehead University, 955 Oliver Road, Thunder Bay, ON P7B 5E1 Canada; 3 Clean Air Metals, 1004 Alloy Drive, Thunder Bay, ON P7B 6A5 Canada; 4 AGC GeoConsulting, 777 Red River Road, Thunder Bay, ON P7B 1J9 Canada; 5 Earth Sciences, Memorial University, 230 Elizabeth Avenue, St. John’s, NL A1C 5S7 Canada; 6 DEL Exploration, Paradise, Paradise, NL A1L 1V6 Canada

**Keywords:** Current deposit, Ni–Cu–PGE, Base-metal sulfide chemistry, S isotopes

## Abstract

The Current deposit is hosted by serpentinized peridotite that intruded rocks of the Quetico Subprovince in the Midcontinent Rift, and is subdivided into three morphologically distinct regions — the shallow and thin Current–Bridge Zone in the northwest, the deep and thick 437–Southeast Anomaly (SEA) Zone in the southeast, and the thick Beaver–Cloud Zone in the middle. The magma parental to the Current deposit became saturated in sulfide as a result of the addition of external S from at least two sources — a deep source characterized by high Δ^33^S (< 3‰) values, and a shallow source, potentially the Archean metasedimentary country rocks, characterized by low Δ^33^S (< 0.3‰). Variations in Δ^33^S–S/Se–Cu/Pd values indicate that the contamination signatures were largely destroyed by interaction of the sulfide liquid with large volumes of uncontaminated silicate melt. The intrusion crystallized sequentially, with the Current–Bridge Zone crystallizing first, followed by the Beaver–Cloud Zone, and lastly by the 437–SEA Zone. This, along with the elevated Cu/Pd ratios in the 437–SEA Zone, which formed as a result of sulfide segregation during an earlier saturation event, and development of igneous layering in this zone, suggests that it represents the feeder channel to the Current deposit. After the intrusion crystallized, the base-metal sulfide mineralogy was modified by circulation of late-stage hydrothermal fluids, with pyrrhotite and pentlandite being replaced by pyrite and millerite, respectively. This fluid activity mobilized metals and semi-metals, including Fe, Ni, S, Se, Co, Cu, Ag, and As, but did not affect the PGE. This contribution highlights the importance of the interplay between magma dynamics and magmatic–hydrothermal processes in the formation of Ni–Cu–PGE-mineralized deposits.

**Supplementary Information:**

The online version contains supplementary material available at 10.1007/s00126-023-01193-9.

## Introduction

Magmatic Ni–Cu–platinum-group element (PGE) sulfide deposits form through a series of suprasolidus processes that have been relatively well constrained and which can largely be summarized by four major events — i) generation of a mafic–ultramafic magma via partial melting of the mantle, ii) saturation of a mantle-derived magma in sulfide and segregation of an immiscible sulfide liquid, iii) interaction of the sulfide liquid with the silicate melt and enrichment of metals in the former based, and iv) concentration of the metal-enriched sulfide liquid to form an ore body (Naldrett 2010). While these fundamental processes are commonplace in magmatic Ni–Cu–PGE deposit, the mechanism by which sulfide saturation occurs varies between deposits. Although several mechanisms have been proposed, including closed-system fractional crystallization, magma mixing, increasing magma *f*O_2_, and addition of externally derived Si or S (Robertson et al. 2015b), most of these, apart from direct addition of S, were deemed incapable of generating economic concentrations of Ni–Cu–PGE mineralization (Ripley and Li 2013). Notable examples of conduit-type Ni–Cu–PGE sulfide deposits that formed via assimilation of distinct contaminants include Norilsk, which was contaminated by anhydrite (e.g., Ripley et al. 2010), Voisey’s Bay, which was contaminated by paragneiss (e.g., Ripley et al. 2002), and Jinchuan, which was contaminated by carbonate rock (e.g., Lehmann et al. 2007). Additionally, contamination by geochemically distinct assimilants within a single conduit system has been described in the Eastern Gabbro of the Coldwell Complex (Midcontinent Rift System), in which the Marathon deposit assimilated Archean sedimentary rock and the northern deposits likely assimilated metamorphosed igneous rock (Shahabi Far et al. 2018; Brzozowski et al. 2020). Regardless of how these fundamental processes operate, the sulfide liquid that results eventually crystallizes to the commonly observed assemblage of pyrrhotite–pentlandite–chalcopyrite (Craig and Kullerud 1969; Kullerud et al. 1969).

It is well understood, however, that subsolidus processes have the potential to modify the mineralogy of the base-metal sulfide (BMS) assemblage, as well as the grade and tonnage of the mineralized system (Prichard et al. 2013; Holwell et al. 2017; Brzozowski et al. 2020; Lawley et al. 2020; Wang et al. 2021). These processes, therefore, have the potential to affect the economic value of a mineralized system (Holwell et al. 2017). An example of a mineralized system whose economic potential is believed to have been improved via subsolidus processes is the Roby Zone of the Lac des Iles deposit in northern Ontario, Canada. Although the mineralization at Lac des Iles is unequivocally magmatic (Barnes and Gomwe 2011; Djon and Barnes 2012; Duran et al. 2016), it has been demonstrated that the Pd enrichment in the Roby Zone was the result of upgrading by magmatic–hydrothermal fluids (Watkinson and Dunning 1979; Hinchey and Hattori 2005). Accordingly, the success of mineral exploration and eventual metal extraction depends on having a strong understanding of these supra- and subsolidus processes and a robust mineral deposit model.

**Fig. 1:**
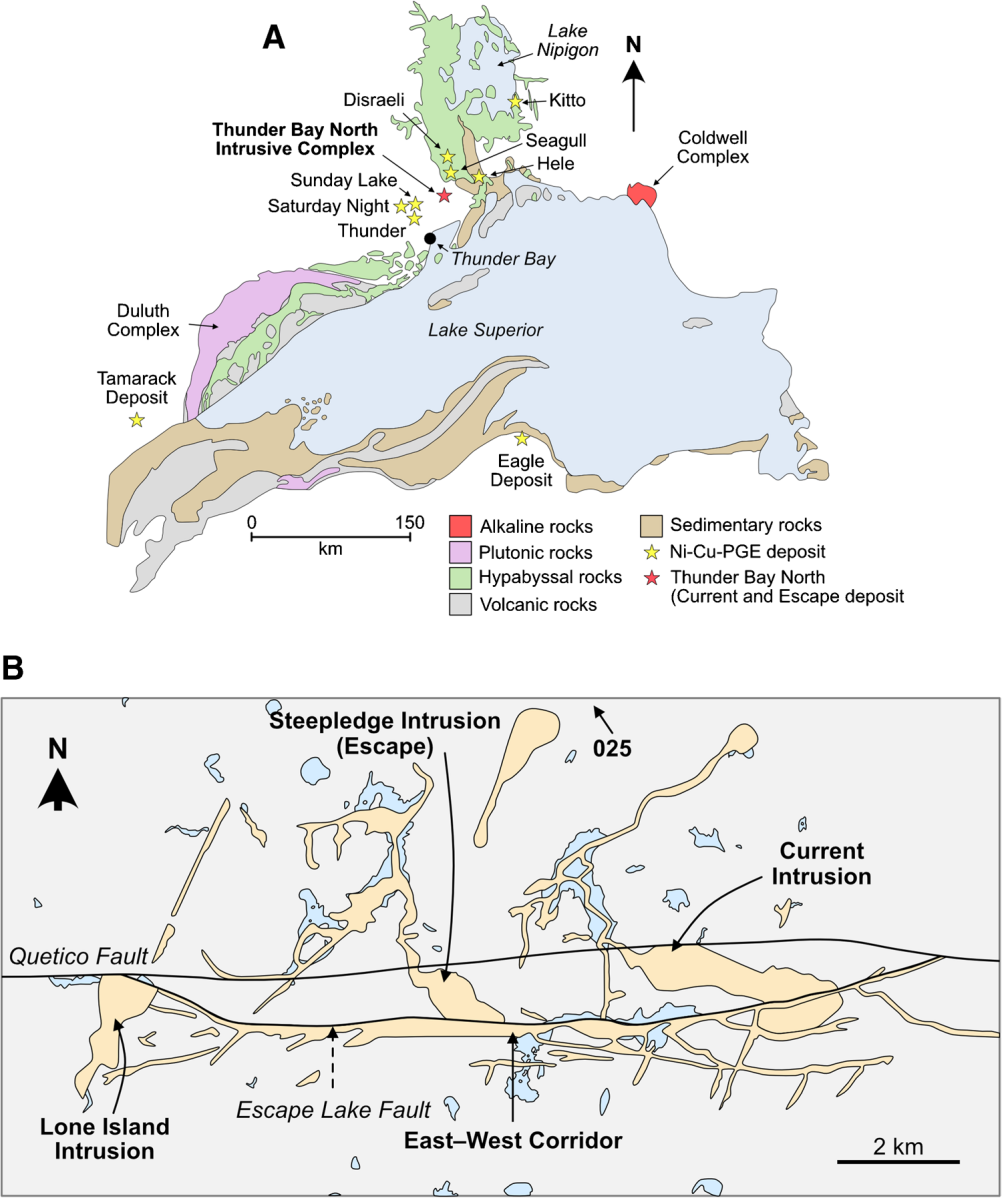
(**A**) Simplified geologic map of the North American Midcontinent Rift illustrating the distribution of rock types and highlighting the location of several Ni–Cu–PGE-mineralized intrusions and complexes, including the Thunder Bay North Intrusive Complex (modified from Good et al. 2015). (**B**) Simplified geologic map showing the locations of mafic–ultramafic intrusions of the Thunder Bay North Intrusive Complex, including the Ni–Cu–PGE-mineralized Current and Escape intrusions (modified from Thomas et al. 2011)

The Thunder Bay North Intrusive Complex (TBNIC) of the Midcontinent Rift System contains a series of mafic–ultramafic intrusions, including the 1,106.6 ± 1.6 Ma Ni–Cu–PGE-mineralized Current and Escape intrusions (Fig. 1B) (Bleeker et al. 2020; Kuntz et al. 2022). Although exploration of these two systems has been ongoing since 2005, limited work has been done to characterize the suprasolidus processes that generated the base- and precious-metal mineralization or the subsolidus processes that modified the mineralization. Accordingly, this contribution focuses on characterizing the suprasolidus processes that generated the BMS mineralization along the length of the Current conduit, as well as the subsolidus processes that modified the initial mineralization, with the ultimate goal of developing a robust mineral deposit model. This is accomplished by integrating detailed petrography, whole-rock and BMS chemistry, and multiple S isotopes (^32^S, ^33^S, ^34^S, ^36^S). This contribution represents one of the first studies to develop a holistic model for a BMS deposit in the Thunder Bay–Nipigon Embayment region. It, therefore, not only has broad implications for the formation of, and exploration for, Ni–Cu–PGE deposits globally, but also lays the foundation for our understanding of deposits in this portion of the Midcontinent Rift System.

## Geological setting of the Thunder Bay North Intrusive Complex

The TBNIC is located in the Quetico Subprovice of the Superior Province in northern Ontario, Canada and represents one of several magmatic Ni–Cu–PGE-mineralized complexes that formed as part of the North American Midcontinent rifting event ca. 1.1 Ga (Fig. 1A) (Bleeker et al. 2020; Kuntz et al. 2022). It comprises several mafic–ultramafic chonoliths that straddle the east–west trending Quetico Fault System (Fig. 1B) (Bleeker et al. 2020). From east to west, these intrusions are the Current, 025, Steepledge, and Lone Island intrusions that are connected by dykes–sills of the East–West Corridor (Fig. 1B) (Kuntz et al. 2022).

**Fig. 2:**
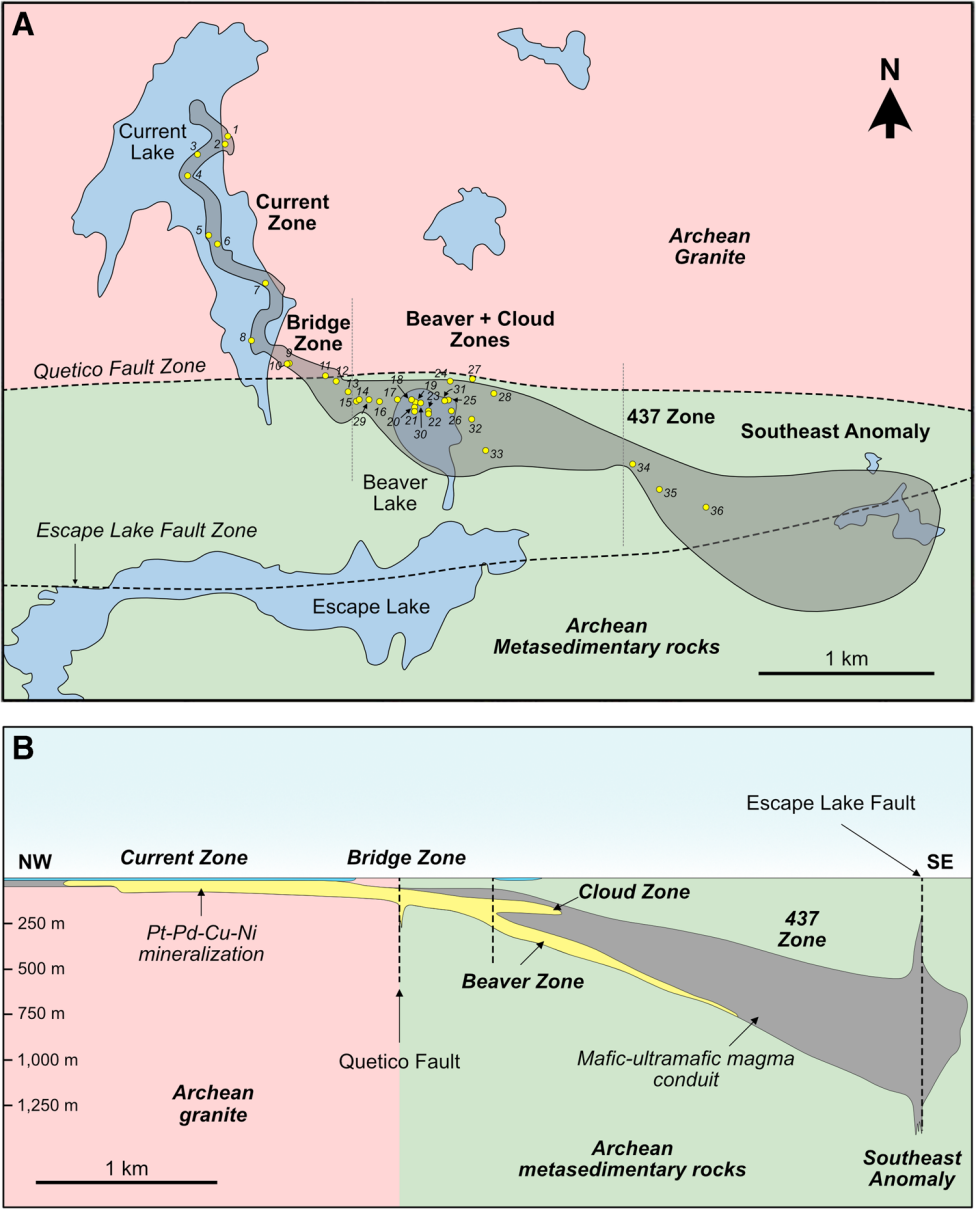
(**A**) Simplified geologic map illustrating the morphology of the Current intrusion (grey) crosscutting the Archean metasedimentary rocks of the Quetico Subprovince (south of Quetico Fault Zone) and Archean granite (north of the Quetico Fault Zone) in plan view, highlighting the relative locations of the five mineralized zones and the Southeast Anomaly (modified from Chaffee 2015). The yellow circles represent the locations of drill holes from which samples were characterized in this study. The grey dashed lines are the UTM locations where the intrusion has been subdivided into the Current–Bridge, Beaver–Cloud, and 437–SEA zones. (**B**) Schematic cross section of the Current intrusion illustrating the change in morphology of the conduit with depth and the relative location of sulfide mineralization in the five zones (modified from Thomas et al. 2011)

The 1,106.6 ± 1.6 Ma Current intrusion has been drilled extensively since 2006 (730 drill holes totaling 162,997 m as of 2020) (Kuntz et al. 2022). It defines a ~ 3.4-km-long northwest–southeast-trending chonolith (Figs. 1B and 2) with a “tadpole-shaped” aeromagnetic anomaly that suggests it extends for up to 6 km towards the southeast (Bleeker et al. 2020); this has been verified by drilling. The chonolith comprises undeformed and unmetamorphosed olivine melagabbro, feldspathic lherzolite, and lherzolite, as well as quartz-bearing gabbro/monzonite (Chaffee 2015; Kuntz et al. 2022).

**Fig. 3:**
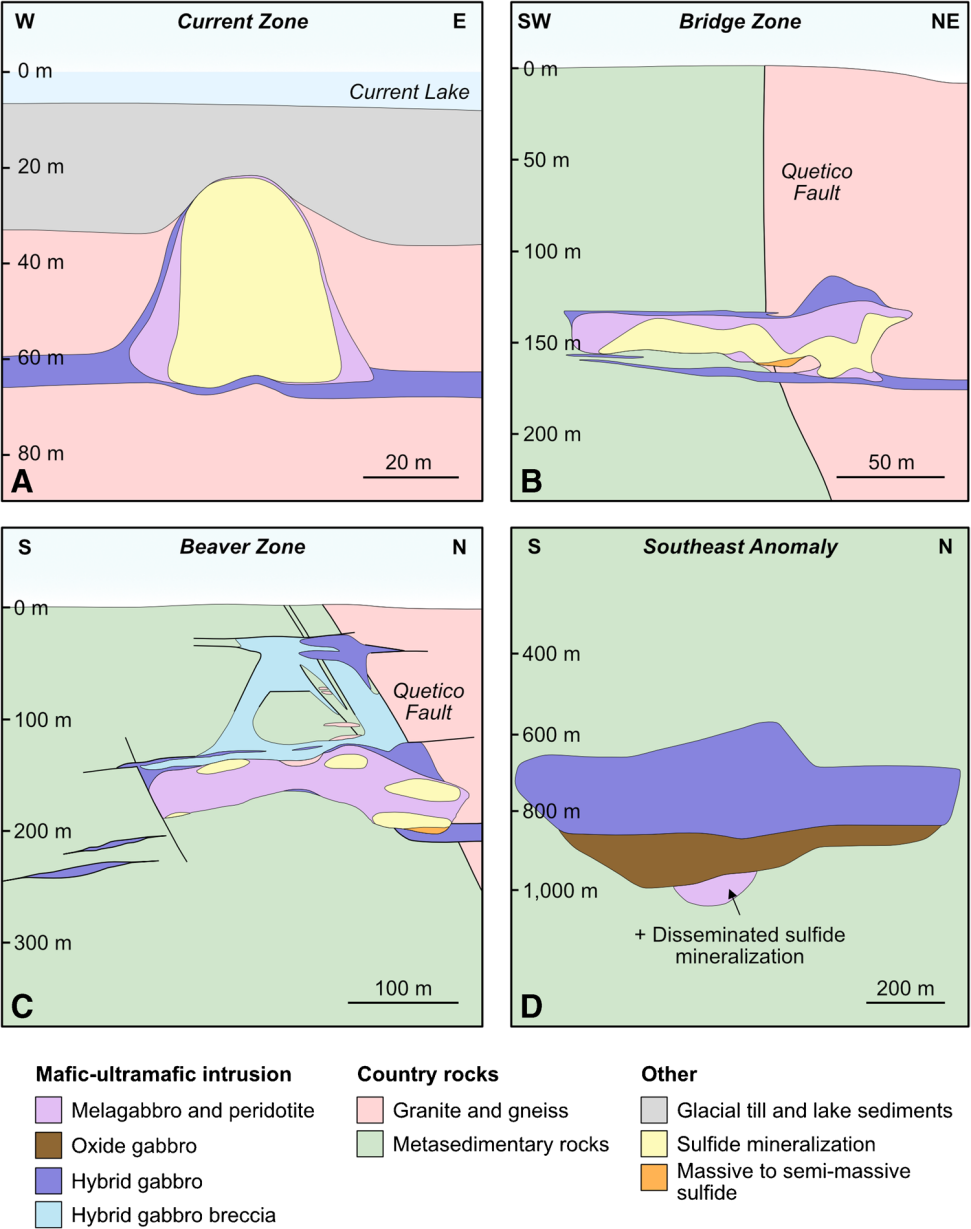
Schematic cross sections of the (**A**) Current Zone, (**B**) Bridge Zone, (**C**) Beaver Zone, and (**D**) Southeast Anomaly illustrating the relationships of the main rock units in the conduit, the change in conduit morphology from northwest to southeast along the intrusion, and the change in location of mineralization that accompanies this change in morphology (modified from Thomas et al. 2011; Bleeker et al. 2020)

The Current chonolith has been subdivided into i) the Current–Bridge Zone to the north, ii) the 437 Zone–Southeast Anomaly (SEA) to the south, and iii) the Beaver–Cloud Zone in between (Fig. 2a). The Current–Bridge Zone is hosted by granite, whereas the Beaver–Cloud and 437–SEA zones are hosted by metasedimentary country rock (Fig. 2) (Kuntz et al. 2022). The lithology of the Current–Bridge and Beaver–Cloud zones largely comprises peridotite (Fig. 3A–C), whereas the 437–SEA Zone is layered from a lower peridotite to melagabbro to an upper oxide gabbro and capped with a quartz-bearing gabbro/monzonite (Figs. 3D, ESM 1S1) (Heggie et al. 2015). Northwest of the Quetico Fault in the Current Zone, the chonolith is thin (< 70 m), sinuous, tubular, shallow (< 60 m to its base), and relatively flat lying (Figs. 2B and 3A) (Kuntz et al. 2022). Straddling the Quetico Fault in the Bridge Zone, the chonolith changes to a more stubby, tabular morphology with a gentle southeast plunge to a depth of < 150 m (Figs. 2B and 3B). From the Beaver Zone to the SEA, the chonolith progressively thickens from ~ 150–500 m, is tabular in shape, and extends to progressively greater depths of up to ~ 1,000 m at the base of the SEA (Figs. 2B and 3C, D).

The BMS mineralization in the Current deposit is hosted within the lherzolite and olivine melagabbro (Fig. 3) (Kuntz et al. 2022). This mineralization largely occurs as disseminated pyrrhotite, pentlandite, chalcopyrite, pyrite, and cubanite, with overall abundances ranging from a few percent to > 25%; small bodies of semi-massive to massive sulfide occur at the base of the Bridge and Beaver zones (Fig. 3B, C) (Bleeker et al. 2020; Kuntz et al. 2022). Base-metal sulfides are distributed throughout the chonolith in the Current Zone and the northwest portion of the Bridge Zone, but become bottom loaded in the southeast portion of the Bridge Zone where it meets the Beaver Zone (Fig. 3A–C). Southeast of the Beaver Zone, the majority of BMSs are bottom loaded. The SEA and Cloud Zone are exceptions to these general characteristics, the former containing only minor BMS, and the latter comprising < 1% finely disseminated and irregularly dispersed chalcopyrite ± pyrrhotite at the roof of the chonolith (Fig. 2B).

## Samples and methods

The mineralogy and texture of BMS were characterized in 284 thin sections collected along the length of 36 drill holes that intersected the Current, Bridge, Beaver, Cloud, and 437 zones, as well as the SEA (Fig. 2A). Five samples of the granite country rock and two samples of the metasedimentary country rock were also characterized (Fig. 2A). Mineral Liberation Analysis (MLA) was conducted on ten thin sections at the CREAIT facility at Memorial University of Newfoundland using an FEI Quanta 400 scanning electron microscope (SEM) equipped with a Bruker XFlash energy dispersive X-ray (EDX) detector and mineral liberation analysis software. Operation conditions of the instrument are provided in the Electronic Supplementary Material (ESM 1).

Bulk-rock geochemical data comprise assays for base metals (Cu), precious metals (Pd, Pt, Ir), and S that were used by Clean Air Metals to define the Current deposit. The vast majority of the assay data were determined at ALS (~ 98% of the database), with the remaining ~ 2% determined at Accurassay Laboratories. Quality assurance and control for these analyses were performed throughout the exploration process to meet the requirements of the National Instrument 43–101. All bulk-rock data utilized herein represent rocks with Pd > 0.01 ppm. Details regarding the analytical methods used to collect the bulk-rock data are provided in the Electronic Supplementary Material (ESM 1)

The trace-element contents of BMS from 65 polished thin sections and pucks from 31 drill holes were determined at the Element and Heavy Isotope Analytical Laboratory at the University of Windsor using an Agilent 7900 ICP–MS coupled with a 193-nm excimer laser. The accuracy of the measured concentrations was assessed by comparing the measured values of sulfide reference UQAC FeS-1 (University du Québec à Chicoutimi, Canada) and MASS-1 to the working values; the measured values are in good agreement with the working values (ESM 2 Table S1). Details regarding instrument operating conditions, standardization, correction of metal argide interferences, and signal processing are provided in ESM 1.

The S isotope composition (^32^S, ^33^S, ^34^S, ^36^S) of BMS was determined in situ using a CAMECA IMS 1280 secondary ion mass spectrometer at the Centre for Microscopy, Characterisation and Analysis, University of Western Australia. Details regarding instrument operating conditions, standardization, and data processing are provided in ESM 1. Sulfur isotope values are reported in delta notation as permil deviations from Vienna Canyon Diablo troilite. Mass-independent fractionation was assessed by calculating the deviation of the measured values from mass-dependant fractionation (Δ^33^S = δ^33^S – 1,000 × [(1 – δ^34^S)^0.515^ – 1], Δ^36^S = δ^36^S – 1,000 × [(1 – δ^34^S)^1.91^ – 1]). Accuracy of the sample measurements was assessed by measuring the isotopic composition of the Nifty-b chalcopyrite, VMSO pentlandite, Alexo pyrrhotite, and Sierra pyrite standards. Their measured compositions are in excellent agreement with the reference values provided by LaFlamme et al. (2016) (ESM 2 Table S2).

## Results

### Bulk-rock geochemistry

**Fig. 4:**
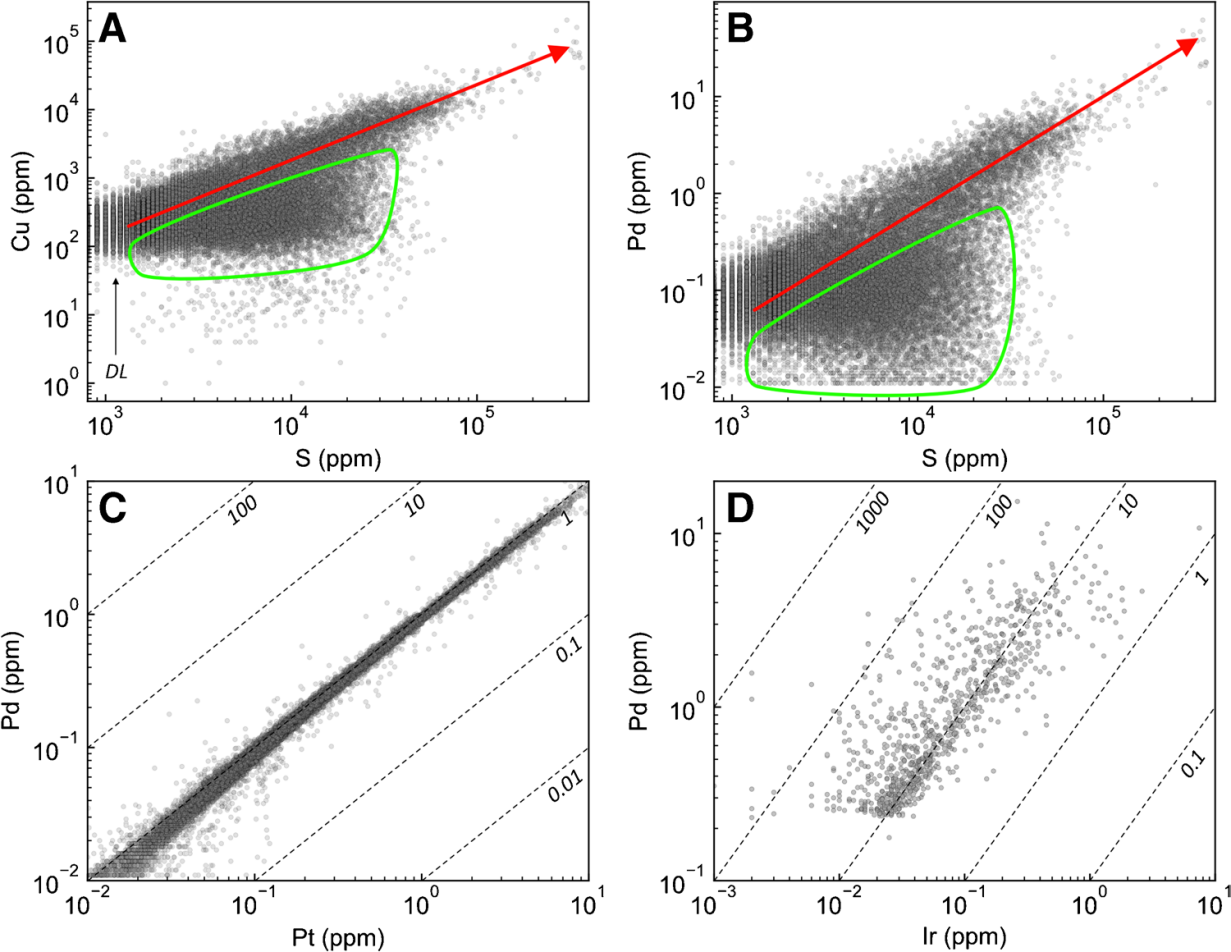
Binary diagrams illustrating the variation in bulk-rock (**A**) Cu–S, (**B**) Pd–S, (**C**) Pd–Pt, and (**D**) Pd–Ir. The red arrow highlights data that exhibits a positive trend on these diagrams, whereas the green field highlights data that falls off this trend. Dashed lines represent constant Pd/Pt and Pd/Ir ratios. DL = detection limits

In metal–S space, Cu and PGE generally correlate positively with S concentrations, but there is a subgroup within which chalcophile elements exhibit no discernable correlation with S (Fig. 4A, B). Similarly, when comparing the Pt-group PGE (PPGE) and Ir-group PGE (IPGE), Pd correlates positively with both Pt (Pd/Pt = 0.9 ± 0.4, average ± 2SD; Fig. 4C) and Ir (Pd/Ir = 21 ± 131; Fig. 4D).

### Base-metal sulfide mineralogy

**Fig. 5:**
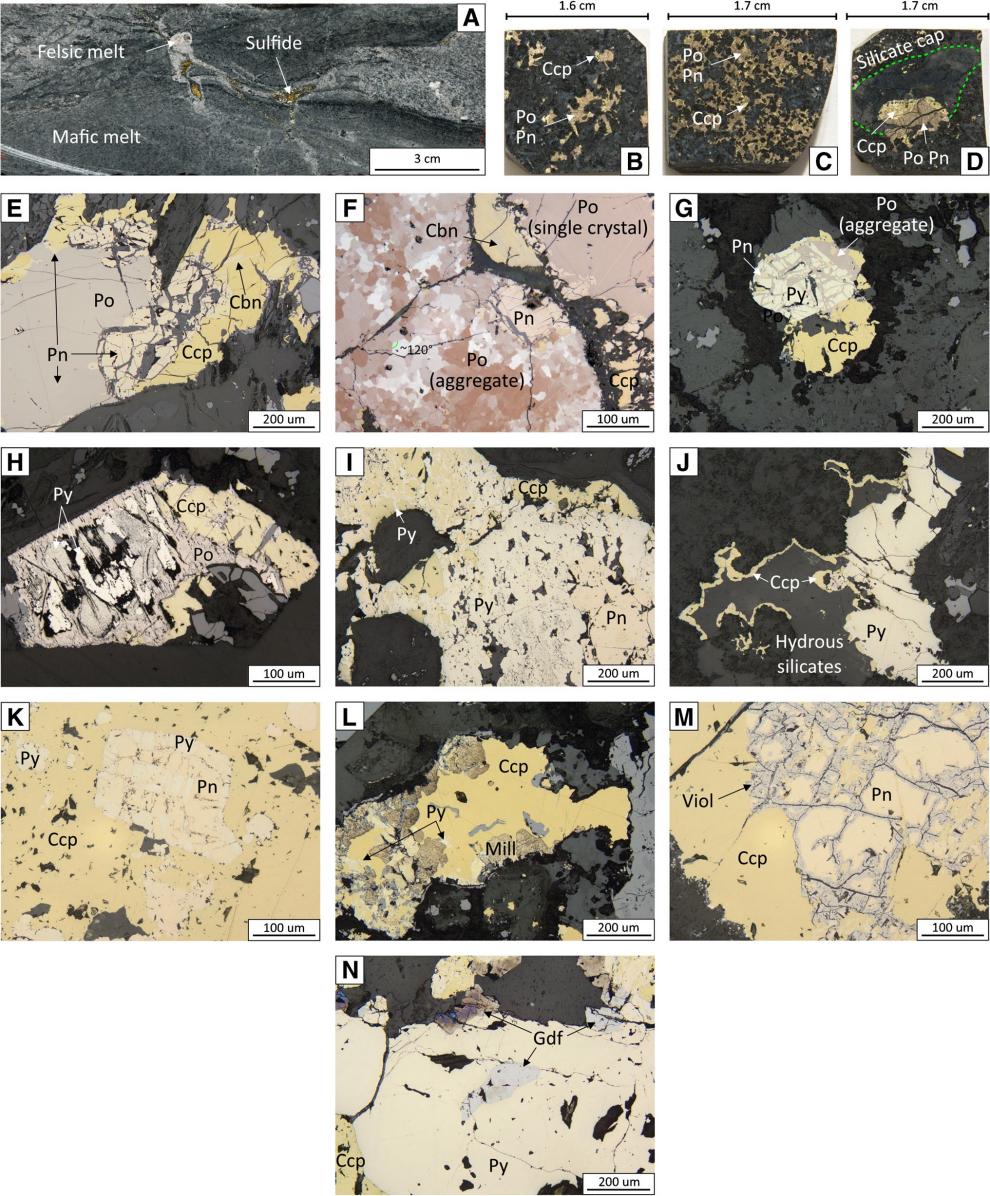
Images of drill core samples (**A–D**) and reflected–light photomicrographs (**E–N**) illustrating representative examples of base-metal sulfides and textures in the Current deposit. (**A**) Mingling between the mafic Current magma and a felsic melt. Note the occurrence of base-metal sulfides where the two magmas mingled. (**B**) Disseminated, (**C**) net-textured, and (**D**) blebby base-metal sulfide mineralization. (**E**) An equilibrium (magmatic) assemblage comprising pyrrhotite–pentlandite–chalcopyrite–cubanite. (**F**) Cross-polarized, reflected-light photomicrograph illustrating pyrrhotite occurring as a single crystal and as an aggregate of multiple crystals. Note the 120° dihedral angles between crystals in the latter. (**G**) Pyrite partially replaced by pyrrhotite. (**H**) Pyrrhotite partially replaced by pyrite. (**I**) An assemblage comprising chalcopyrite–pentlandite–pyrite. (**J**) Chalcopyrite restricted to an alteration patch and physically associated with pyrite. (**K**) An assemblage of chalcopyrite–pentlandite–pyrite in which the pyrite was partially replaced by pentlandite. (**L**) An assemblage of chalcopyrite–millerite–pyrite. (**M**) An assemblage comprising largely chalcopyrite–pentlandite, with violarite occurring along fractures in pentlandite. (**N**) An assemblage of chalcopyrite–pyrite–gersdorffite. Po = pyrrhotite, Pn = pentlandite, Ccp = chalcopyrite, Cbn = cubanite, Py = pyrite, Mill = millerite, Viol = violarite, Gdf = gersdorffite

The samples that were characterized for this study comprise variably mineralized mafic–ultramafic rocks from the chonolith, granitic country rocks, and samples where the two have mingled (Fig. 5A). Base-metal sulfides are largely disseminated (Fig. 5B), but can be net-textured (Fig. 5C) and blebby (Fig. 5D). The latter sulfide variety can be mineralogically segregated, with a portion comprising pyrrhotite–pentlandite and portion comprising chalcopyrite (Fig. 5D); a fine-grained assemblage of silicate minerals commonly surrounds the chalcopyrite (Fig. 5D).

The BMS assemblages throughout the Current intrusion comprise variable proportions of pyrrhotite, pentlandite, chalcopyrite, and pyrite, with lesser cubanite, millerite, violarite, and rare troilite and gersdorffite. Chalcopyrite, pyrrhotite, and pentlandite largely occur as equilibrium assemblages with sharp, curved contacts (Fig. 5E). Pyrrhotite can occur as single large (mm-sized) crystals (Fig. 5E, F) or as aggregates of numerous fine-grained (tens of microns) crystals that share ~ 120º angles; these textural varieties of pyrrhotite can occur within the same thin section (Fig. 5F). Troilite occurs as wavy intergrowths in pyrrhotite. Pentlandite can occur as large (hundreds of microns) crystals associated with chalcopyrite–pyrrhotite assemblages (sometimes at the contact between the two), as flames within pyrrhotite (Fig. 5E), and in veinlets that crosscut pyrite (similar to pyrrhotite in Fig. 5G). Cubanite occurs as laths within chalcopyrite (Fig. 5E, F).

Pyrite generally occurs in disequilibrium with pyrrhotite (Fig. 5H), as large (mm-sized) crystals either associated with chalcopyrite–pentlandite–cubanite or isolated from other BMS, and as wormy intergrowths in chalcopyrite (Fig. 5I). Rarely, chalcopyrite associated with pyrite can occur isolated within alteration patches (Fig. 5J). Locally, pyrite can occur as blocky intergrowths with pentlandite (Fig. 5K). Millerite occurs as aggregates of irregular crystals in chalcopyrite; it is most common in samples that contain pyrite (Fig. 5L). Violarite occurs along fractures in pentlandite (Fig. 5M). Gersdorffite occurs as anhedral–euhedral grains within chalcopyrite, pyrite, and silicates (Fig. 5N).

**Fig. 6:**
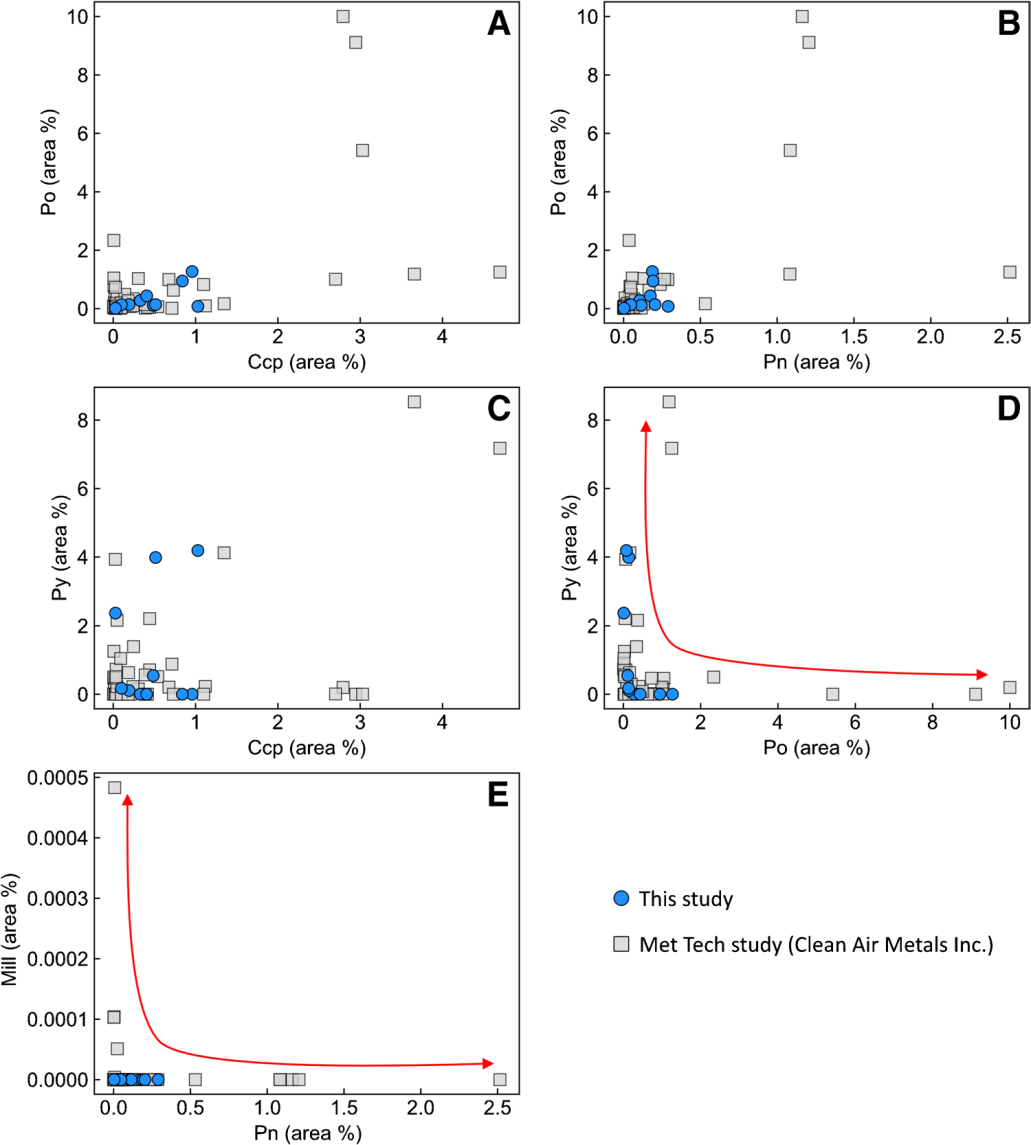
Binary diagrams illustrating the variation in abundances of (**A**) chalcopyrite–pyrrhotite, (**B**) pentlandite–pyrrhotite, (**C**) chalcopyrite–pyrite, (**D**) pyrrhotite–pyrite, **(E**) pentlandite–millerite obtained by mineral liberation analysis. Note the strong negative, non-linear correlation between the abundances of pyrrhotite–pyrite and pentlandite–millerite. The grey squares are data from an unpublished metallurgical study by Clean Air Metals Inc

The visual proportion of pyrite to other BMS varies significantly within the sample population, with some samples having no pyrite, others being composed entirely of chalcopyrite–pyrite, and no pyrrhotite, and others comprising pyrrhotite–pentlandite–chalcopyrite–pyrite–millerite. Mineral abundance data obtained via MLA on ten samples confirm this thin section-scale variability in sulfide mineralogy (Fig. 6). Of note is that the abundances of pyrrhotite and pyrite exhibit a clear negative and non-linear correlation (Fig. 6D). This mineralogical variability matches that obtained on a larger sample population by Clean Air Metals (grey squares in Fig. 6). Within this larger sample set, it is also evident that a similar negative and non-linear correlation exists between pentlandite and millerite (Fig. 6E).

### Base-metal sulfide trace-element chemistry

#### Distribution among BMS

**Fig. 7:**
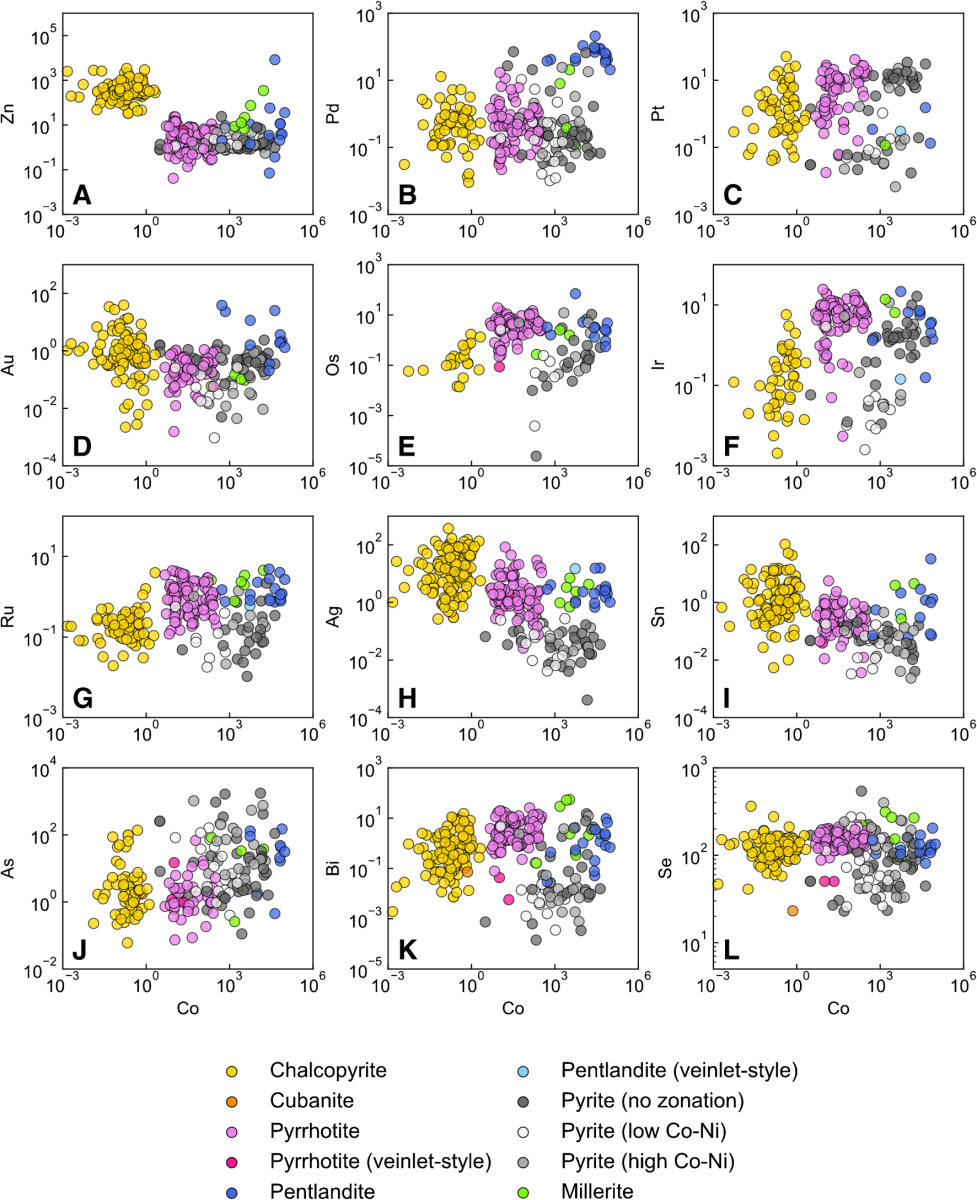
Binary diagrams illustrating the variation in metal (**A–H**) and semi-metal (**I–L**) concentrations in base-metal sulfides as a function of Co concentration. All concentrations are in ppm

The trace-element chemistry of the six most common BMS throughout the Current deposit is provided in ESM 2 Table S3 and illustrated in Fig. 7. In the Current deposit, the order of decreasing Co contents is pentlandite = millerite > pyrite > pyrrhotite > chalcopyrite–cubanite (Fig. 7A). Iron-rich BMS consistently have lower Zn concentrations than Cu-rich BMS (Fig. 7A). Palladium, Pt, and Au exhibit similar ranges of concentration (up to three orders of magnitude) that are generally indistinguishable among the BMS, apart from pentlandite, which has consistently elevated Pd contents (up to 208 ppm), and some pyrite that extend to elevated Pd values similar to pentlandite (Fig. 7B–D). The concentrations of the IPGE (Os, Ir, Ru) among the BMS are indistinguishable and exhibit similar ranges (e.g., four orders of magnitude for Ir; Fig. 7E–G). Silver and Sn exhibit similar distributions among the BMS, with their concentrations generally decreasing in the order chalcopyrite > pyrrhotite–pentlandite–millerite > pyrite (Fig. 7H, I). The concentration of As typically extends to higher values in pyrite compared to chalcopyrite–pyrrhotite–pentlandite; Bi exhibits a mirrored distribution (Fig. 7J, K). The concentration of Se in chalcopyrite, pyrrhotite, pentlandite, and millerite is similar and exhibits relatively limited variability, whereas its concentration in pyrite extends to notably lower values than in the other BMS (Fig. 7L).

#### Cu/Pd and S/Se values

**Fig. 8:**
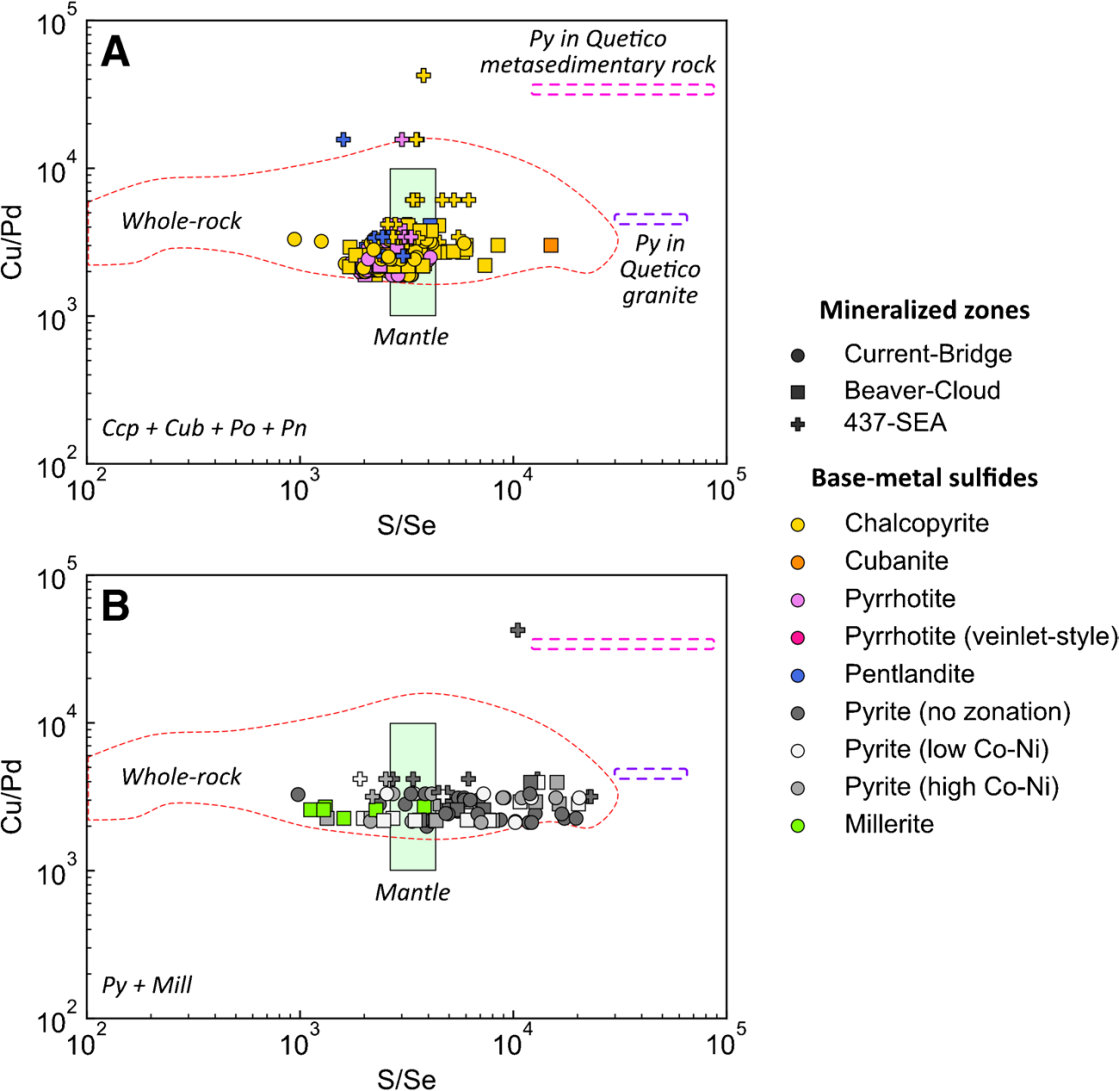
Binary diagrams illustrating the variation in bulk-rock Cu/Pd and sulfide S/Se in (**A**) the primary chalcopyrite–cubanite–pyrrhotite–pentlandite assemblage and (**B**) the secondary pyrite–millerite assemblage. The red, dashed field outlines the range of bulk-rock Cu/Pd and S/Se values. The purple and pink dashed fields represents the bulk-rock Cu/Pd ratio of Archean granitic and metasedimentary country rock and S/Se of its pyrite (n_Py_ = 2 and n_Py_ = 4, respectively); data for metasedimentary pyrite is from Caglioti (2023). The mantle ranges for Cu/Pd (1,000–10,000) and S/Se (2,632–4,350) are from Barnes et al. (1993, 2015b), and Eckstrand and Hulbert (1987) and Palme and O’Neil (2014), respectively

Bulk-rock Cu/Pd ratios of samples from the Current–Bridge (1,892–3,275) and Beaver–Cloud (1,902–4,100) zones are largely indistinguishable, exhibit limited variability, and fall entirely within the range of mantle values (Fig. 8). The 437–SEA Zone has consistently higher and more variable Cu/Pd ratios (3,166–42,444, Fig. 8).

The S/Se values of chalcopyrite (942–8,480), pyrrhotite (1,731–7,158), and pentlandite (1,597–4,076) are indistinguishable and will be considered together (Fig. 8A). Pyrite and millerite are considered separately as they are secondary BMS. Based on the primary BMS assemblage, the S/Se values of the Current–Bridge (942–5,880), Beaver–Cloud (1,706–15,023), and 437–SEA (1,597–6,180) zones overlap and are generally indistinguishable (Fig. 8A). These S/Se values extend to lower and higher values than those of the mantle (Fig. 8A). Pyrite and millerite in the Current deposit are characterized by S/Se values that are notably more variable than the primary BMS assemblage, ranging from 979 to 23,091, and extend to values notably lower and higher than the mantle range (Fig. 8B). Pyrite from a granite country rock sample with Cu/Pd of 4,500 has S/Se values of 30,066–64,426 (Fig. 8). Pyrite from two metasedimentary country rock samples with Cu/Pd of 34,000–59,000 have S/Se values of 12,848–82,249 (Fig. 8).

### Multiple sulfur isotopes

**Fig. 9:**
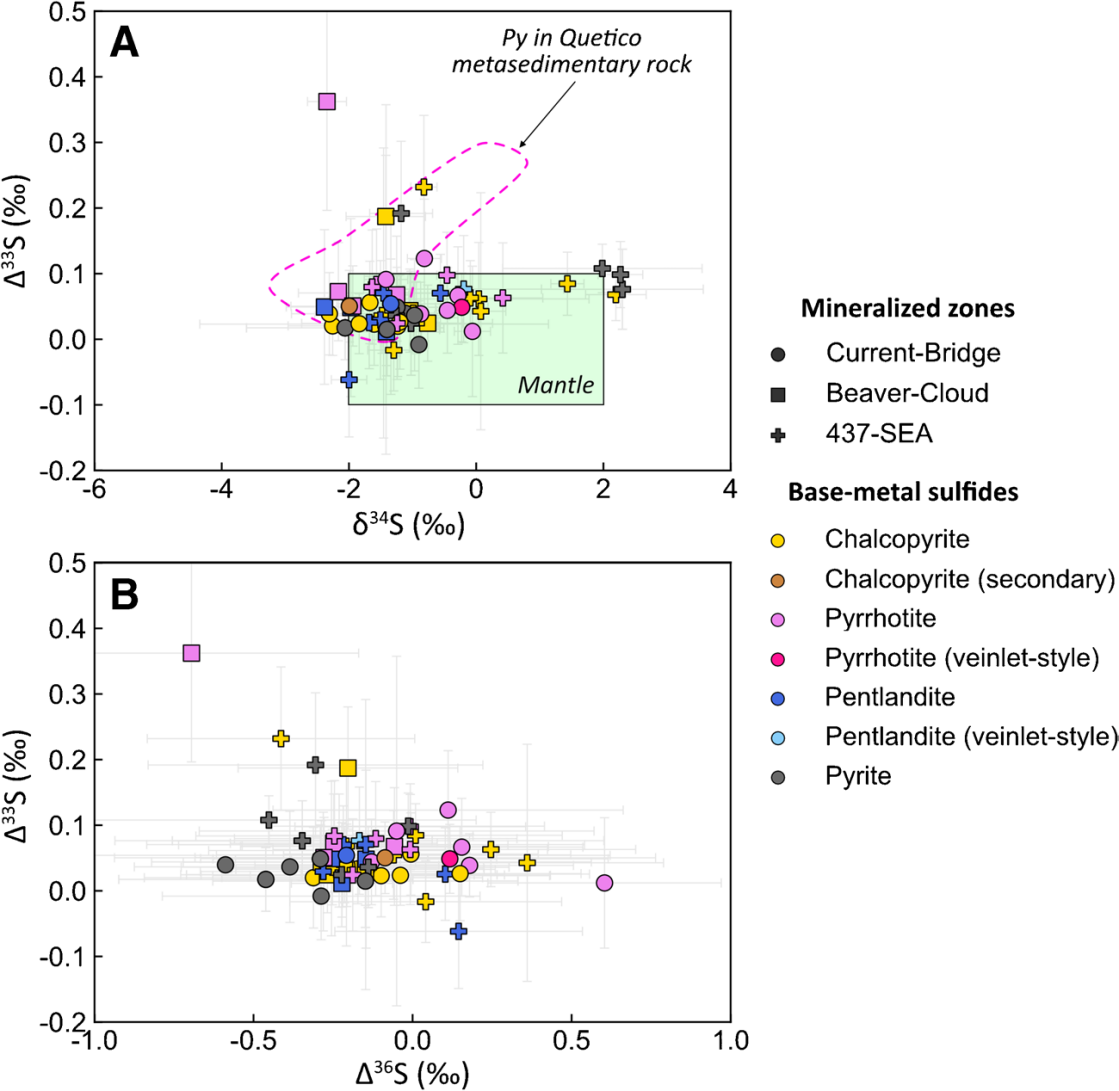
Binary diagrams illustrating the variation in (**A**) Δ^33^S–δ^34^S and (**B**) Δ^33^S**–**Δ^36^S of base-metal sulfides. The mantle range for Δ^33^S is from Farquhar (2002) and Bekker et al. (2009), and for δ^34^S is from Lesher and Burnham (2001) and Ripley and Li (2003). The mantle ranges for Cu/Pd (1,000–10,000) and S/Se (2,632–4,350) are the same as in Fig. 8. The pink, dashed field highlights the composition of pyrite from Quetico metasedimentary rocks from Caglioti (2023). Error bars for S isotopes are 2σ

The overall S isotope composition of the Current deposit (-0.06‰ to 0.36‰ Δ^33^S and -2.4‰ to 2.30‰ δ^34^S, ESM 2 Table S4) largely falls within the range of mantle values, although several samples have Δ^33^S values that are distinctly higher than mantle (Fig. 9A). The overall S isotope compositions of the BMS, both primary and secondary, are indistinguishable (Fig. 9A, B). Similarly, the S isotope compositions of BMS in the Current–Bridge and Beaver–Cloud zones are also largely indistinguishable (Fig. 9A, B). Notably, there is no significantdifference in the S isotope composition of texturally distinct BMS (Fig. 5B–D) or those associated with mafic magma–granite magma mingling (Fig. 5A) (ESM 2 Table S4). In Δ^33^S–Cu/Pd–S/Se space, BMS generally exhibit horizontal trends, with large variability in isotope composition and limited variability in Cu/Pd and S/Se values, apart from a few samples from the 437–SEA Zone with elevated Cu/Pd, and pyrite with elevated S/Se values (ESM 1 Figs. S3A, B).

## Discussion

### Origin of the pyrite–millerite assemblage in the Current deposit

Although pyrite and millerite can form via magmatic processes by recrystallization from monosulfide solid solution (MSS), this only occurs in sulfide liquids characterized by elevated S/metal ratios (approximately 40 wt. % S at 600 °C) (Naldrett et al. 1967; Kullerud et al. 1969; Craig 1973). Most sulfide liquids, however, do not achieve such high S/metal ratios, and so primary pyrite in magmatic sulfide deposits is quite rare (Piña et al. 2016). Formation of magmatic millerite is largely restricted to Ni-rich komatiitic ores, whereas those that occur in mafic–ultramafic systems are largely the result of low-temperature alteration of pentlandite (Barnes et al. 2011; Duran et al. 2015). Given these physicochemical constraints, the occurrence of pyrite as anhedral, pitted grains (Fig. 5H–L) rather than the euhedral grains expected for primary pyrite (e.g., Dare et al. 2011; Duran et al. 2015; Piña et al. 2016), the disequilibrium textures exhibited in pyrite–pyrrhotite (Fig. 5H) and pentlandite–millerite assemblages, the patchy chemical zonation in pyrite (ESM 1 Fig. S2E), and the inverse correlation between the abundance of pyrrhotite–pyrite and pentlandite–millerite (Fig. 6D, E), it is likely that the pyrite and millerite in the Current deposit are not magmatic in origin, but rather formed by the low-temperature (< 230 °C; Naldrett and Kellurud 1967; Naldrett et al. 1967; Craig 1973; Misra and Fleet 1974) alteration of pyrrhotite and pentlandite, respectively. This characterization will be used throughout the discussion when characterizing magmatic and hydrothermal processes.

### Sulfide saturation and metal enrichment–depletion

Many primary BMS in the Current deposit are characterized by mantle S/Se values (2850–4350; Eckstrand and Hulbert 1987; Palme and O’Neil 2014), but significant variability towards values greater and lower than mantle is also observed (Fig. 8A). Likewise, although BMS at Current are characterized by Δ^33^S–δ^34^S values that are largely within range of mantle values (Δ^33^S = 0 ± 0.1‰, δ^34^S = 0 ± 2‰; Lesher and Burnham 2001; Farquhar 2002; Ripley and Li 2003; Bekker et al. 2009), several BMS have Δ^33^S that are distinctly higher than the mantle (Fig. 9A, D, F). Although variations in R factor alone can explain the lower-than-mantle S/Se and mantle-like Δ^33^S values (ESM 1 Fig. S4), it cannot explain the elevated S/Se and Δ^33^S values. These elevated values must, therefore, represent the addition of S from an external source. Given the mantle-like δ^34^S for all of the BMS and elevated Δ^33^S (Fig. 9), it seems likely that the contaminant was Archean in age as these isotopic signatures are diagnostic of Archean sedimentary reservoirs (Farquhar and Wing 2003, 2005).

The country rocks provide a potential local source of S as they comprise Archean metasedimentary, and granitic rocks of the Quetico Subprovince (Hart and MacDonald 2007). Although there is limited S isotopic data on these country rocks, the data that are available for the Archean Quetico metasedimentary rocks demonstrate that it is heterogeneous, with δ^34^S values in the range of -3.03‰ to 0.61‰ and Δ^33^S values in the range of 0.01‰ to 0.29‰ (Caglioti 2023). Considering this heterogeneity, the fact that the Quetico Subprovince represents a mixture of crustal sources (Williams 1991), and the significant variability of Δ^33^S during the Archean (Farquhar et al. 2010), it is possible that the Quetico metasedimentary rocks contain S with highly variable and positive Δ^33^S values. Similarly, there is no isotopic data available for the Archean granitic country rocks into which the northern portion of the Current intrusion intruded, but the lack of deviation of δ^34^S–Δ^33^S from mantle values of sulfides hosted within a mixture of mafic magma and felsic country rock melt (Fig. 5A, ESM 2 Table S3) suggests that the granitic country rocks do not contain a significant mass independent fractionation (MIF) signal. Lastly, it is also possible that S was sourced from Archean rocks at depth, which has been suggested for other Ni–Cu–PGE-mineralized intrusions in the Midcontinent Rift System, including the Marathon deposit (Shahabi Far et al. 2018; Brzozowski et al. 2020, 2021) and Duluth Complex (Ripley et al. 2007).

It has been well documented that contamination signatures recorded by S isotopes and S/Se values can be diluted by interaction of the contaminated sulfide liquid with uncontaminated pulses of silicate melt (Lesher and Burnham 2001; Ripley and Li 2003; Hiebert et al. 2013, 2016; Queffurus and Barnes 2015; Smith et al. 2016, 2021; Shahabi Far et al. 2018; Brzozowski et al. 2020, 2021), with complete destruction of the geochemical–isotopic signatures at R factors > 1,000 (ESM 1 Fig. S4). Considering the importance of R factor to the enrichment of sulfide liquids in metals in Ni–Cu–PGE deposits, particularly volumetrically small, conduit-type, PGE-rich systems like Current (Figs. 2 and 3), it is possible that the largely mantle-like S isotope and S/Se values of BMS that extend towards non-mantle values may be the product of dilution caused by variably high R factors of < 10,000. To assess this, the S isotope and S/Se values of BMS, and bulk-rock Cu/Pd values are compared to compositions modeled using the R factor equations of Ripley and Li (2003).

Copper, Pd, and Se concentrations are modelled following the closed-system R factor equation of Ripley and Li (2003) and employing sequential steps of batch equilibration:

1 $${C}_{{sul}_{f}}^{metal}=\frac{{C}_{{sul}_{i}}^{metal}+(R* {C}_{si{l}_{i}}^{metal})}{1+ \frac{R}{{D}_{sul-sil}^{metal}}}$$

The parameters ${C}_{{sul}_{i}}^{metal}$ and ${C}_{{sul}_{f}}^{metal}$ are the initial and final concentrations of a metal in the sulfide liquid, respectively, ${C}_{si{l}_{i}}^{metal}$ is the initial concentration of a metal in the silicate melt, $R$ is the incremental R factor (i.e., the R factor of each individual pulse of magma), and ${D}_{sul-sil}^{metal}$ is the sulfide liquid–silicate melt partition coefficient for a given metal. Sulfur isotopes are modeled using the open-system R factor equation (Ripley and Li 2003):

2 $${dS}_{su{l}_{f}}=\frac{{dS}_{su{l}_{i}} + {R}^{o}\left({dS}_{si{l}_{i}} + {\Delta S}_{sul-sil}\right)}{1+ {R}^{o}}, where\;{R}^{o}= \frac{{C}_{sil}^{S}}{{C}_{sul}^{S}}*R$$

The parameters ${dS}_{su{l}_{i}}$ and ${dS}_{su{l}_{f}}$ correspond to the initial and final (after sulfide liquid–silicate melt interaction) S isotope compositions of the sulfide liquid, respectively, ${dS}_{si{l}_{i}}$ is the initial S isotope composition of the incoming silicate melt, ${\Delta S}_{sul-sil}$ is the S isotope fractionation factor between sulfide liquid and silicate melt, and ${C}_{sil}^{S}$ and ${C}_{sul}^{S}$ are the concentrations of S in the silicate melt and sulfide liquid, respectively. Values used for the model parameters are provided in Table 1, along with an explanation of why the values were chosen. Given the likelihood that the contaminant was Archean in age and the overall mantle-like δ^34^S values of the samples (Fig. 9), modeling focused on Δ^33^S–S/Se–Cu/Pd. Because the nature of the Archean contaminant is not known, modeling was done using several contaminant starting compositions representing assimilation of S with a range of Δ^33^S values from mantle-like (0.1‰), to those similar to metasedimentary pyrite in the Quetico Subprovince (up to 0.3‰; Caglioti 2023), to those notably higher than mantle (1‰, 3‰, and 10‰); these values are within the range of Δ^33^S values of Archean sedimentary reservoirs (Farquhar and Wing 2003, 2005; Farquhar et al. 2010).

**Table 1:** Parameters for numerical modeling

	Value	Note	Reference
Silicate melt			
Cu_sil.i_	100	Similar to the values of the theoretical parental melt to the Current deposit.	Heggie (2012)
Pd_sil.i_	0.01		
Cu/Pd_sil.i_	10000		
S_sil_	400	Similar to the median S content of unmineralized dikes and volcanic rocks in the Midcontinent Rift north of Lake Superior.	Cundari et al. (2021)
Se_sil.i_	0.10	Calculated by maintaining a mantle S/Se ratio of 4,000.	Brzozowski et al. (2021)
S/Se_sil.i_	4000	Within the range of mantle values (2,632-4,350).	Eckstrand and Hulbert (1987), Palme and O’Neil (2014)
Δ^33^S_sil.i_	0	Within the range of mantle S isotope values.	
Sulfide liquid			
Cu_sul.i_	80000	Within the range of metal tenors in the Current deposit. Calculated using assay data and the method of Kerr (2003).	Kerr (2003)
Pd_sul.i_	25		
S_sul_	360000	Sulfur content similar to pyrrhotite-pentlandite-chalcopyrite.	
Se_sul.i_	10	Within the range of bulk-rock values of other Ni–Cu–PGE deposits globally. Minimum value required to generate the high S/Se ratios observed in the Current sulfides. Does not significantly affect the model at high R factors (>1,000).	Queffurus and Barnes (2015)
S/Se_sul.i_	36000	Serves as the starting contaminated composition.	
Δ^33^S_sul.i_ (Model 1)	0.1	Model in which the contaminant has a mantle-like Δ^33^S value.	
Δ^33^S_sul.i_ (Model 2)	0.3	Model in which the contaminant has a Δ^33^S value similar to sedimentary pyrite of the Archean Quetico Subprovince.	Caglioti, unpublished MSc thesis
Δ^33^S_sul.i_ (Model 3)	1	Models in which the contaminant has Δ^33^S values heavier than mantle.	Farquhar and Wing (2003, 2005), Farquhar et al. (2010)
Δ^33^S_sul.i_ (Model 4)	3		
Δ^33^S_sul.i_ (Model 5)	10		
Other parameters			
R	100		
R^o^	0.11		
ΔS_sul-sil_	0.00		Ripley and Li (2003)
D^Cu^_sul-sil_	2130	Maximum sulfide liquid-silicate melt partition coefficients.	Barnes and Ripley (2016)
D^Pd^_sul-sil_	536000		
D^Se^_sul-sil_	2339		

All concentrations are in ppm. S isotopes are in permille

**Fig. 10:**
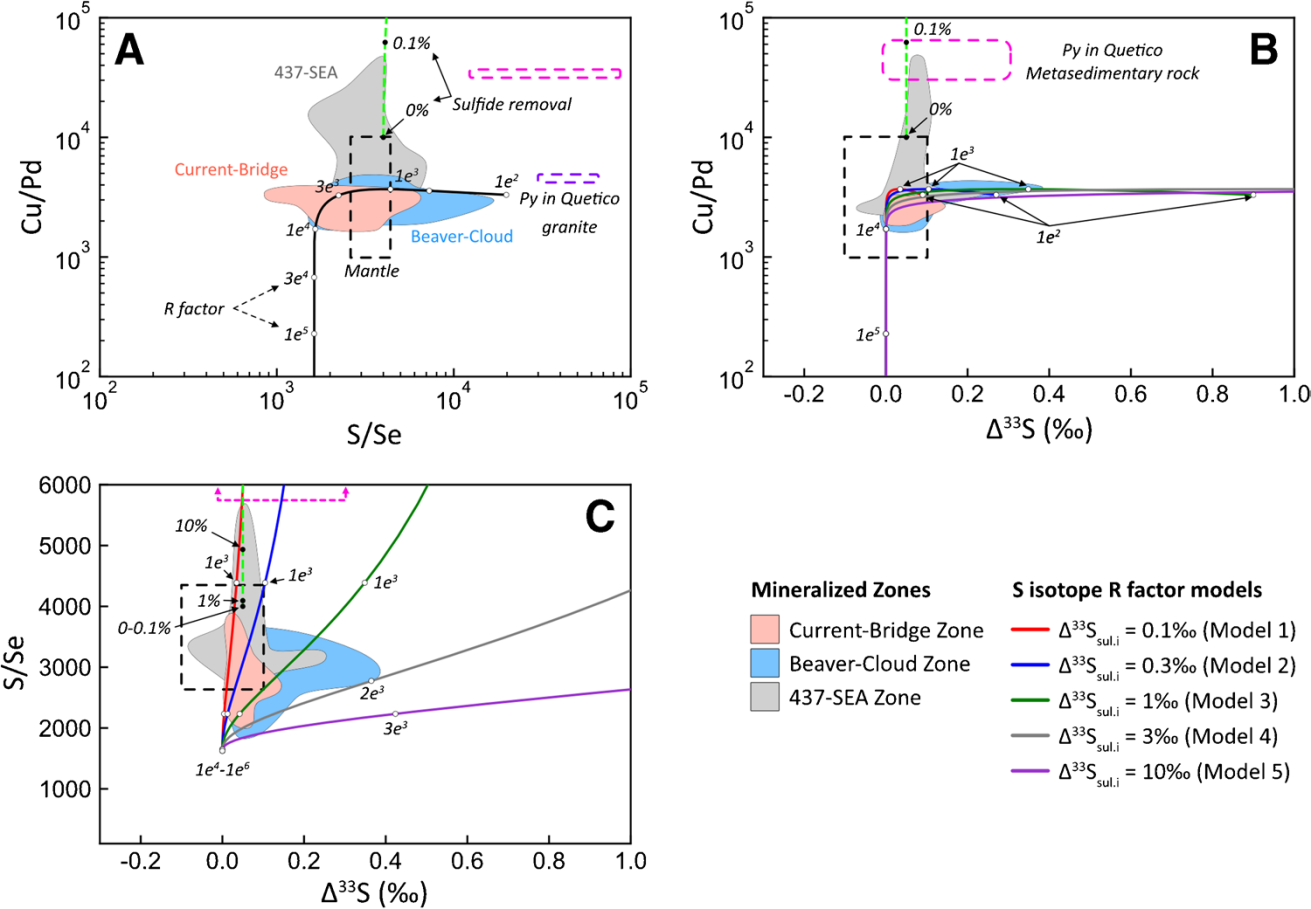
Binary diagrams illustrating the modeled variations in (**A**) S/Se–bulk-rock Cu/Pd, (**B**) Δ^33^S–bulk-rock Cu/Pd, and (**C**) Δ^33^S–S/Se as a function of variable R factor and contamination by rocks with different Δ^33^S values (colored, solid lines). The green, dashed line represents modeled compositional variations as a function of sulfide liquid removal. The colored fields represent the S/Se ratio of primary chalcopyrite–cubanite–pyrrhotite–pentlandite in the different mineralized zones and bulk-rock Cu/Pd. References for the mantle ranges are the same as those in Fig. 9. The purple and pink dashed fields represents the bulk-rock Cu/Pd ratio of Archean granitic and metasedimentary country rock and S/Se of its pyrite; data for metasedimentary pyrite is from Caglioti (2023). The numbers on the model curves represent R factor and the degree of sulfide liquid removal

**Fig. 11:**
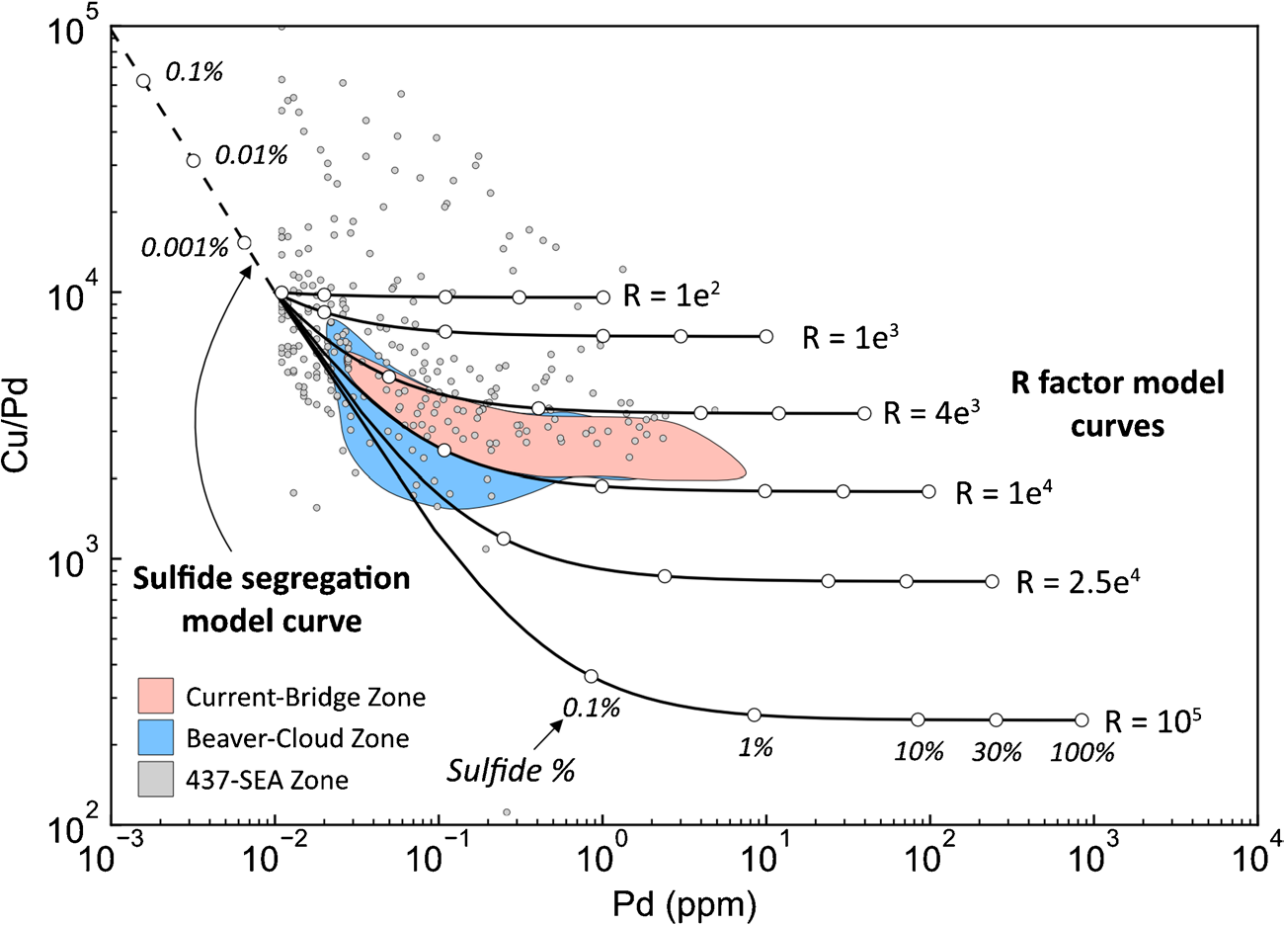
Binary diagram illustrating the variation in bulk-rock Cu/Pd and Pd in the Current deposit. The colored fields represent the distribution of data for the Current–Bridge and Beaver–Cloud zones, whereas the data points represent the 437–SEA Zone. The solid curves represent modeled variations as a function of R factor and sulfide abundance. The dashed curve represents modeled variations as a function of sulfide liquid removal. The Cu–Pd contents of the starting melt and the sulfide liquid–silicate melt partition coefficients are provided in Table 1. The numbers on the curve represent sulfide percent, either as the amount present in the rock (in the case of the R factor models) or the amount of sulfide liquid removed (in the case of the sulfide segregation model)

According to the model, as R factor increases from 100 to ~ 10,000, the S/Se value of the sulfide liquid decreases more rapidly than the Cu/Pd ratio, whereas at R factors > 10,000, the opposite is observed, with S/Se being essentially invariable and Cu/Pd decreasing significantly (Fig. 10A). The Cu/Pd–S/Se data from the Current deposit generally follows the model trend at R factors < 10,000, exhibiting limited variability in Cu/Pd values, but significant variability in S/Se values (Fig. 10A). This suggests that i) the sulfide liquid at Current experienced R factors < 10,000, consistent with R factors estimated from bulk-rock Cu and Pd (Fig. 11), and ii) the silicate melt assimilated material that was characterized by elevated S/Se values. Based on the modeling, the minimum S/Se value of the contaminant was likely in the range of 36,000, which is within the range of values of pyrite in the Archean Quetico granitic (30,066–64,426, ESM 2 Table S3) and metasedimentary country rocks (12,848–82,249; Caglioti 2023).

The modeled variations in Cu/Pd–Δ^33^S exhibit similar trends for all of the hypothetical contaminants, with significant variability in Δ^33^S at R factors < 10,000, and essentially no variability in Δ^33^S at R factors > 10,000 (Fig. 10B), similar to S/Se. The model curves for the various contaminants in S/Se–Δ^33^S space exhibit distinct trends based on how positive the Δ^33^S value of the contaminant is, with the overall slope of the curves increasing with isotopically lighter contaminants (Fig. 10C). Two things are evident from the model curves and distribution of Current data. First, it seems unlikely that the contaminant(s) had Δ^33^S values greater than 3‰ as the Current data have S/Se values notably higher than those predicted by models using such contaminants (Fig. 10C). Second, none of the contaminant models individually can explain the distribution of BMS compositions at Current, with some BMS having mantle-like Δ^33^S, but elevated S/Se values, and others having high Δ^33^S, but mantle-like S/Se values (Fig. 10C). The elevated Cu/Pd values of the 437–SEA Zone (Fig. 10A, B) indicate that the silicate melt(s) from which they crystallized must have lost < 0.1% of their sulfide liquid prior to or during intrusion (Fig. 10A, B). It is possible that the elevated S/Se values in this zone are also the result of sulfide removal rather than contamination. This scenario seems unlikely, however, as generating S/Se values in the range of 6,000 would require removal of > 10% sulfide liquid from the melt, which would have severely depleted the melt in Pd (Fig. 10). Rather, the distribution of data suggests that the Current magma(s) assimilated externally derived S from isotopically distinct reservoirs, some of which had mantle-like Δ^33^S and others that had high Δ^33^S values of up to 3‰ (Fig. 10C).

The MIF signal recorded by some BMS in the Current–Bridge and Beaver–Cloud zones (Fig. 10C), therefore, likely originated from the addition of S with elevated Δ^33^S from Archean rocks at depth. In contrast, the mantle-like Δ^33^S and elevated S/Se values that largely characterize BMS in the 437–SEA Zone likely record assimilation of S from the local metasedimentary country rocks, which exhibit similar geochemical–isotopic signatures (Fig. 10C). This is consistent with the fact that the 437–SEA Zone was one of the final zones to have remained magmatically active in the Current intrusion, as evidenced by the lithologic layering in this zone that could only have been developed as a result of relatively closed-system fractional crystallization when the magmatic system was waning (ESM 1 Fig. S1) (Heggie et al. 2015). This portion of the conduit would, therefore, have experienced the greatest contact time with the metasedimentary country rocks, increasing the amount of metasedimentary S that was added to the magma.

An implication of this interpretation is that the sulfide liquid formed at depth and was carried upwards, the possibility of which has long been questioned (Lesher 2019). Recent numerical simulations and textural evidence, however, demonstrate that dense sulfide liquids may be transported upwards by attachment to vapor bubbles in shallow, degassing magmas (Yao et al. 2019; Yao and Mungall 2020). Barnes et al. (2019) demonstrated this concept for the Norilsk ores, where they identified segregated sulfide globules capped by highly fractionated, residual silicate melt (i.e., sulfide liquid + vapor bubble). A similar textural variety of sulfide occurs at Current (Fig. 5D), suggesting that the Current magma(s) were degassing during transport, which may have supported the upward movement of sulfide liquid.

### Remobilization of metals

The peridotite host rocks of the Current deposit are pervasively serpentinized. This, along with several lines of mineralogical and geochemical evidence, support the idea that late-stage hydrothermal fluids circulated through the host rocks of the deposit. This evidence includes i) the ubiquity of secondary pyrite and millerite after pyrrhotite and pentlandite, respectively (Fig. 5H–J, L), ii) the occurrence of pyrrhotite and pentlandite veinlets that crosscut secondary pyrite (Fig. 5G), iii) the presence of chalcopyrite restricted to patches of late-stage hydrous minerals (Fig. 5J), iv) the lack of correlation between bulk-rock S and base–precious metals (e.g., Cu and Pd) for a subpopulation of samples from the deposit (Fig. 4A, B), and v) the occurrence of gersdorffite with pyrite (Fig. 5N). Although gersdorffite can crystallize from sulfide liquids (Hem and Makovicky 2004), the generally low As content of the primary BMS at Current (Fig. 7J, ESM 2 Table S3) suggests that the As content of the original sulfide liquid was low, precluding the possibility of gersdorffite being a magmatic phase. Such a low As content in BMS is consistent with the fact that As is often undersaturated in sulfide liquid unless it was added to the silicate magma via assimilation of an As-rich contaminant (Smith et al. 2021).

Given the presence of pyrite after pyrrhotite (Fig. 5H) and millerite after pentlandite (Fig. 5L), it is clear that at least some amount of Fe and Ni must have been remobilized since pyrrhotite has a higher Fe content than pyrite (~ 62 wt. % vs. ~ 47 wt. %), and pentlandite has a lower Ni and higher Fe content than millerite (~ 34 wt. % vs. ~ 65 wt. % Ni and ~ 33 wt. % vs. 0 wt. % Fe). The replacement of pyrrhotite by pyrite and pentlandite by millerite would, therefore, release Fe to the fluid, whereas the latter reaction would sequester Ni from the fluid; although the source of this additional Ni is uncertain, it could have originated from the olivine that was pervasively serpentinized, as has been demonstrated in the serpentinized Huangshandong Ni–Cu sulfide deposit in northwestern China (Wang et al. 2021). Mobilization of Fe and Ni is also consistent with the occurrence of pyrrhotite and pentlandite veinlets that crosscut pyrite (Fig. 5G), implying that this textural variety of pyrrhotite and pentlandite must have crystallized from fluids that circulated after the replacement of primary pyrrhotite by pyrite, as well as the occurrence of gersdorffite (Fig. 5N). Although removal of Fe can sufficiently explain the replacement of pyrrhotite by pyrite, it is likely that S was also added during this reaction. This is evident by the lack of correlation between bulk-rock S and metals in some samples from the Current deposit (Fig. 4A, B), indicating that S was likely mobile.

Considering that pyrite has a relatively invariable S content of ~ 53 wt. % and the Se content of pyrrhotite exhibits relatively limited variability (50–223 ppm), the large range in S/Se values exhibited by pyrite (Fig. 8B) cannot be entirely the result of S addition during pyrrhotite replacement as this would result in a maximum S/Se value in pyrite of 10,600 (530,000 ppm S / 50 ppm Se), whereas pyrite in the Current deposit exhibits values as high as 64,000 (Fig. 8B). Accordingly, some Se must have been released from pyrrhotite to the fluid as it was replaced by pyrite; this is consistent with the greater range in Se content of pyrite that extends to notably lower values (as low as 23 ppm) than the majority of pyrrhotite (Fig. 7L). Given that Se is thought to be most mobile in acidic, saline, and oxidizing fluids (Prichard et al. 2013), this implies that the fluids that reacted with and replaced pyrrhotite and pentlandite by pyrite and millerite, respectively, had low pH, high salinity, and high *f*O_2_. Importantly, these are also conditions under which several metals may be mobilized, including some of the PGE (Mountain and Wood 1988; Pan and Wood 1994; Wood 2002; Hanley et al. 2005; Liu and McPhail 2005), although recent studies suggest these strict conditions may not be a requirement for metal mobilization (Sullivan et al. 2022).

Despite favorable fluid conditions for the mobility of the PPGE, the range of concentrations of Pd and Pt, as well as Au, in pyrrhotite and pyrite are largely indistinguishable, implying that the replacement reaction did not add or remobilize these metals (Fig. 7B–D). This is consistent with the positive correlations between bulk-rock Pd–Pt (Fig. 4C) and Pd–Ir (Fig. 4D). The IPGE (Ir, Os, and Ru) extend to lower concentrations in pyrite compared to pyrrhotite (Fig. 7E–G). Although these lower concentrations may result from IPGE remobilization by a hydrothermal fluid, large-scale remobilization seems unlikely given that the IPGE are generally considered immobile relative to, for example, Pd (Xiong and Wood 2000; Wood 2002; Sullivan et al. 2022). Given the lower concentrations of Sn and Bi in pyrite compared to pyrrhotite, it is likely that the lower IPGE content in pyrite relates to the formation of IPGE–Sn–Bi–Pb-bearing PGM, which are heterogeneously distributed throughout pyrite, as well as other BMS (ESM 1 Fig. S2) and were excluded from integration regions when processing laser ablation spectra. The higher As content of pyrite relative to pyrrhotite (Fig. 7J) suggests that As was added to the system by the hydrothermal fluid that replaced pyrrhotite by pyrite. This is consistent with the presence of gersdorffite (Fig. 5N) and the mobility of As in Ni–Cu–PGE deposits (Gervilla and Kojonen 2002; Le Vaillant et al. 2015, 2016). Similarly, the concentration of Co extends to notably higher values in pyrite relative to pyrrhotite, suggesting that it was also added to the mineralizing system (Fig. 7A). Given that Ag does not generally occur as an independent mineral (apart from electrum), its lower concentration in pyrite relative to pyrrhotite (Fig. 7H) suggests that it was remobilized from the BMS assemblage during replacement of pyrrhotite by pyrite.

Although not related to the replacement of pyrrhotite by pyrite, Cu was also likely remobilized given i) the occurrence of some chalcopyrite within hydrous mineral assemblages (Fig. 5J) (cf. Brzozowski et al. 2020) and ii) the lack of correlation between bulk-rock Cu and S in a subpopulation of samples from the Current deposit (Fig. 4A). This Cu could not have been added to the system, however, as such a process would generate a vertical trend in bulk-rock Cu/Pd vs. Pd space, which is not observed (Fig. 11). Rather, it must have been remobilized within the system.

These results have important implications for the Current deposit because they demonstrate that metals were variably mobilized by late-stage hydrothermal processes. At the deposit scale, the tenors of Cu and Pd were not affected by this event, as is evident by their coherent behavior in Cu/Pd vs. Pd space (Fig. 11). The IPGE tenors were also unaffected by this event, but their hosts were modified, with As-bearing IPGM becoming a host as a result late-stage As addition. The Ni and Co tenors of the deposit were likely improved during hydrothermal circulation given the presence of millerite after pentlandite, the latter of which has a higher Ni content, and the higher concentration of Co in pyrite after pyrrhotite. In contrast, the Ag tenor of the deposit was likely reduced by removal from the mineralizing system as evidenced by its lower concentration in pyrite after pyrrhotite.

### Feeder zone and direction of magma flow

The direction of magma flow, and hence the location of the magma feeder zone, remain topics of contention in the Current deposit, with both SE to NW magma flow and NW to SE magma flow both being suggested (Bleeker et al. 2020). Three physicochemically distinct features of the SEA point towards a model whereby magma flow in the Current intrusion was from SE to NW, suggesting that the SEA could have been the feeder zone to the intrusion.

#### Loss of sulfide liquid

The SEA is the only zone in the Current intrusion to be characterized by Cu/Pd values that are elevated relative to mantle values (Fig. 10A, B), indicating that it is the only zone to have crystallized from Pd-depleted magma that lost sulfide liquid after it had equilibrated with the silicate magma and scavenged metals (Figs. 10 and 11). Given this, and the fact that bulk-rock Cu/Pd ratios throughout the Current deposit show relatively limited variability (Fig. 11), this loss of sulfide liquid was likely a local event, which we suggest is the removal of sulfide liquid via drainage down towards deeper portions of the intrusive system below the SEA. This suggestion is consistent with the lower Pd contents of pentlandite in this zone compared to pentlandite in the other zones of the deposit (ESM 2 Table S3). Such backflow of sulfide liquid towards deeper portions of an intrusive system has been described by Barnes et al. (2015a). Additionally, Pd depletion is also a feature of the feeder zone to the Marathon conduit-type magmatic sulfide deposit (Good et al. 2015). Loss of sulfide liquid requires this region of the deposit to have experienced a period of decreased magmatic activity, otherwise the sulfide liquid would likely have remained in suspension, resulting in mineralization that was similar to the other portions of the Current deposit, with no Pd depletion, but this is not observed. A decrease in magmatic activity would have allowed for more efficient settling of sulfide liquid droplets or percolation of sulfide liquid through pore spaces between silicate minerals, promoting the removal of sulfide liquid from the SEA. In the case of percolation, it was demonstrated by Mungall and Su (2005) that a sulfide liquid would not be capable of percolating through a cumulate pile by capillary forces, but could be forced through pore spaces by the flow of the enclosing silicate melt. Accordingly, if magmatic activity in the SEA decreased, backflow of the magma could have forced the sulfide liquid through grain boundaries, allowing it to percolate through the cumulate pile and to be removed from the SEA. Additionally, maintaining this Pd-depleted signature in the magma from which the SEA crystallized suggests that, after sulfide loss, the metal content of the mineralizing system was not replenished by significant influx of more primitive magma. Together, these metal characteristics require that the Current deposit crystallized in stages, with the SEA crystallizing last. In such a scenario, the SEA would have acted as a relatively independent system in its final stages prior to crystallization, allowing the magma and sulfide liquid in this zone to acquire distinct geochemical signatures resulting from loss of sulfide liquid via gravity drainage, potentially down the feeder zone. An alternative possibility to explain the Pd depletion in the SEA is retention of sulfide liquid in traps at depth. This scenario seems unlikely, however, because these traps would have been upstream of the deposit, resulting in Pd depletion in all of the zones of the Current deposit, which is not observed (Figs. 10 and 11).

Progressive crystallization from northwest to southeast could explain the recrystallized pyrrhotite observed throughout the Current intrusion (Fig. 5F). Deformation could not have caused this recrystallization because the intrusion is undeformed (Kuntz et al. 2022). An alternative explanation for the recrystallization is thermal metamorphism (Good et al. 2015). This would have required two features — i) some portions of the Current intrusion would need to have been significantly crystallized as pyrrhotite must have already been present and it recrystallizes from MSS at temperatures of < 650 °C (Kullerud et al. 1969; Kelly and Vaughan 1983; Ebel and Naldrett 1996; Lusk and Bray 2002), and ii) there must have been a sustained source of heat. Both of these requirements would have been satisfied in the progressive crystallization scenario, with the still magmatically active 437–SEA Zone providing the necessary heat. This direction of progressive crystallization is also consistent with changes in the morphology of the intrusion, with the shallower and thinner Current–Bridge Zone likely having begun crystallizing prior to the deeper and thicker Beaver–Cloud and 437–SEA zones (Figs. 2 and 3).

An alternative possibility to explain the Pd-depleted nature of the SEA is equilibration of the Current magma, flowing from northwest to southeast, with sulfide liquid. If such a scenario were true, then one would expect a systematic depletion in Pd from Current–Bridge to Beaver–Cloud to SEA, but this is not observed (Figs. 10 and 11). Additionally, it is difficult to conceive of a scenario whereby the Current pluton would have intruded from shallower depths (in the northwest) to greater depths (in the southeast). Although magma may migrate along weaknesses in the crust and be emplaced laterally, magma flow is principally upwards, driven by buoyancy and pressure forces (Longo et al. 2023). Given that the Southeast Anomaly occurs at a depth of ~ 1000 m and the Current–Bridge Zone occurs at a depth of ~ 50 m (Fig. 2b), it seems more likely for magma to have flowed from southeast upwards towards the northwest.

#### Local source of S

Base-metal sulfides in the SEA record distinct S isotope signatures compared to BMS in the other zones of the deposit, having Δ^33^S values that are largely within the range of mantle values compared to the Δ^33^S values that are higher than mantle in the other zones (Fig. 10). This indicates that the sources of S that contributed to sulfide saturation in the SEA were different than those which contributed to saturation in the Current–Bridge and Beaver–Cloud zones. Specifically, the magmas from which the latter zones crystallized likely became sulfide saturated by addition of S from a source at depth, whereas S from the local metasedimentary country rocks was also added to the magma from which the SEA crystallized (Fig. 10c). Addition of local S to the magma from which the SEA crystallized, and the lack of evidence for this addition elsewhere in the Current intrusion, can be explained by increased contact time between the SEA magma and the country rock, which would have allowed for greater diffusive transfer of S into the SEA magma (Robertson et al. 2015a; Barnes and Robertson 2019). Although local S addition could also have occurred via bulk assimilation as a result of, for example, erosion of the conduit floor, this seems unlikely as there is no evidence of bulk-rock contamination: i) partially assimilated xenoliths of country rock material are not observed in the SEA, ii) addition of significant amounts of siliceous country rock material would have resulted in the crystallization of orthopyroxene, but orthopyroxene is rare at Current, and iii) rocks throughout the Current intrusion, including the SEA, have Th/La ratios that are systematically lower than mantle values (0.07–0.1; Chaffee 2015; Yahia et al. 2022). Local addition of S only in the SEA implies that the flow of the SEA magma was restricted to the southeast portion of the Current intrusion, which is hosted by metasedimentary country rock, and/or that the magma was relatively stagnant in this area. It is likely that both scenarios occurred for two reasons. First, sequential crystallization of the Current intrusion from northwest to southeast would have progressively isolated the SEA from the other portions of the conduit, restricting magma flow to portions of the conduit hosted by metasedimentary country rock. Second, the Pd-depleted nature of the SEA (i.e., high Cu/Pd ratios) requires that the magma from which it crystallized lost sulfide liquid, which requires the SEA to have experienced a period of decreased magmatic activity that would have promoted gravitational settling. This decreased magmatic activity would have allowed the SEA magma to stagnate in contact with the metasedimentary country rock. Considering that the Pd content of pentlandite is lower in the SEA compared to the other zones in the Current deposit (ESM 2 Table S3), it is likely that this local addition of S triggered a separate sulfide saturation event in the SEA magma during and/or after the removal of earlier-formed sulfide liquid. The removal of earlier-formed sulfide liquid would have depleted the magma in Pd, as well as other metals, as demonstrated by the Rayleigh fractionation model in Figs. 10 and 11, such that the locally formed sulfide liquid was depleted in Pd, now recorded as Pd-depleted pentlandite.

#### Lithological layering

The above geochemical–isotopic characteristics that are unique to the SEA (elevated bulk-rock Cu/Pd, elevated S/Se ratios in sulfides, and mantle-like Δ^33^S values in sulfides) suggest that this zone was potentially the final portion of the Current conduit to have crystallized. This interpretation is supported by the lithologic layering and accompanied changes in major–minor-element chemistry along drillhole that is observed only in this zone (Heggie et al. 2015). From base to top, this layering comprises peridotite, melagabbro, upper oxide gabbro, and quartz-bearing gabbro/monzonite, and is accompanied by systematic decreases in MgO–Cr and increases in Al_2_O_3_ up drillhole (ESM 1 Fig. S1). Such layering in the SEA, which is also observed in other conduit-type systems (e.g., Kabanga, Uitkomst, Eastern Gabbro; Gauert et al. 1995; Li et al. 2002; Maier et al. 2011; Cao et al. 2019), could have formed via closed-system fractional crystallization (Heggie et al. 2015) or open-system processes, such as slumping and saltation. Although it is difficult to distinguish between closed- vs. open-system processes with the available data, we suggest that the layering was the result of essentially closed-system processes for three reasons. First, the systematic changes in major–minor-element chemistry from the peridotite to the quartz-bearing gabbro/monzonite mimic the geochemical variation expected during fractionation of a magma. Second, there is a lack of rhythmic layering in the SEA, which may be expected if the layering formed by repeated magma replenishment followed by crystallization on the wall rock and collapse (Shaw 1997). Lastly, there is no evidence in drill core for folding of the lithologic layers, which may be expected if the layering formed as a result of slumping (Shaw 1997). We, therefore, suggest that the series of rock types that make up the SEA were the result of magmatic fractionation. This process occurred once the energy of the magmatic system waned and the conduit was no longer being replenished by magma.

Taken together, i) the Pd-depleted nature of the magma from which the SEA crystallized, ii) the distinct source of S that contributed to sulfide saturation in this zone, and iii) the lithological layering observed in this zone indicate that the SEA was the final zone to have crystallized and is, therefore, the feeder to the Current intrusion. This indicates that magma flow in the Current intrusion was from southeast to northwest.

### Deposit model for the Current Ni–Cu–PGE system

**Fig. 12:**
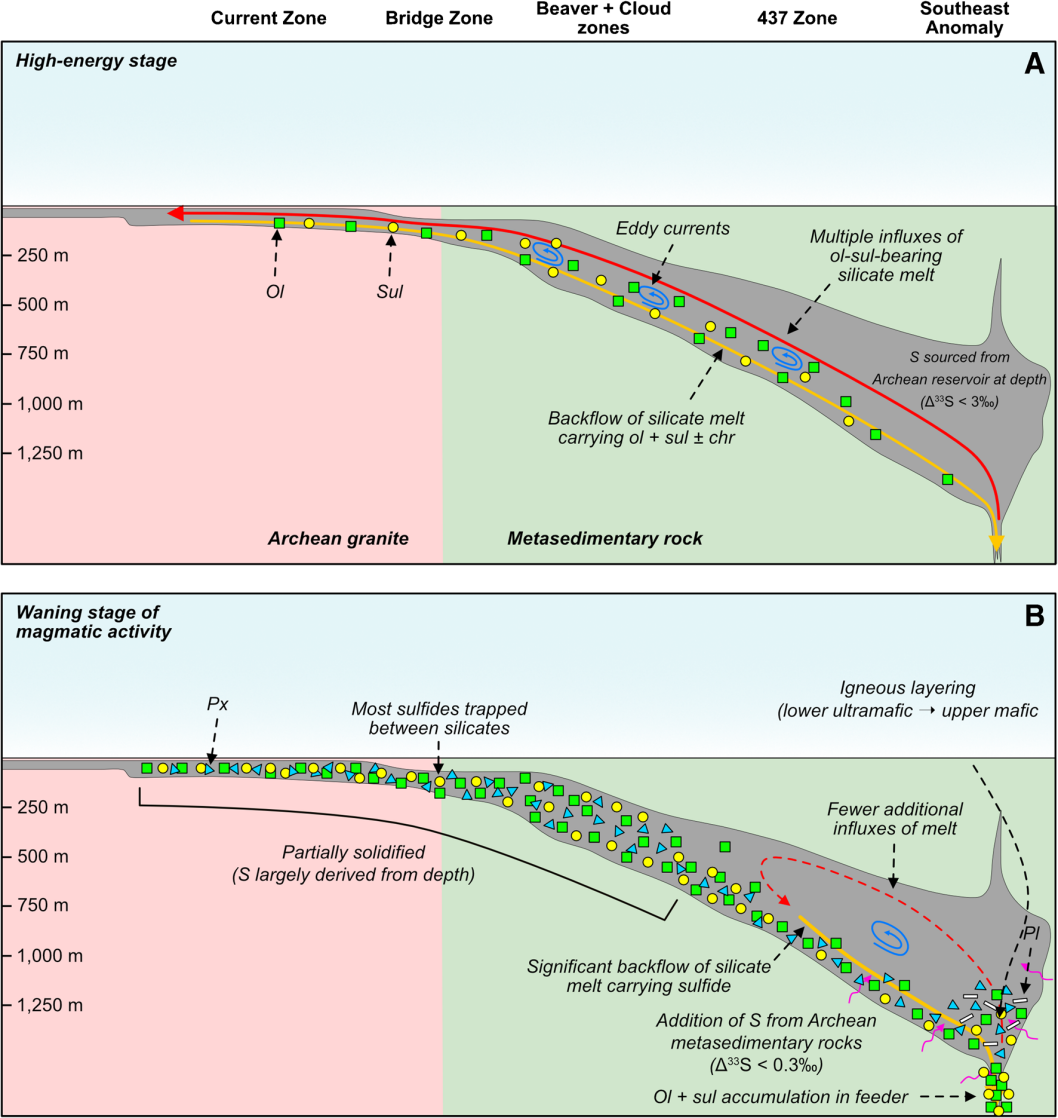
Two-stage schematic model illustrating the processes that led to the formation of sulfide liquid and its subsequent enrichment in metals in the Current deposit — (**A**) high-energy stage and (**B**) waning stage of magmatic activity

We have developed a holistic mineral deposit model for the Current Ni–Cu–PGE deposit that incorporates the mineralogical–geochemical–isotopic features characterized here with the physical features of the intrusion. This model highlights the key mineralizing processes that operated throughout the intrusion to generate the mineralization, as well as how these processes changed as the mineralizing system evolved. Consider a shallow-dipping conduit that was emplaced at shallow levels into Archean metasedimentary and granitic country rocks. During the early, high-energy stage of system development, the conduit was charged with multiple pulses of olivine- and sulfide-laden magma (Fig. 12A). The sulfide liquid carried into the conduit formed at depth by the addition of S from Archean rocks and was entrained upwards, perhaps facilitated by the presence of vapor bubbles (Fig. 5C), and was initially characterized by elevated S/Se and Δ^33^S (up to 3‰) values. During intervals of magmatic quiescence, the magma flowed down the conduit, carrying with it some of the olivine and sulfide liquid that it introduced (Fig. 12A). This process of influx and backflow could have generated eddy currents, aiding the entrained sulfide liquid in interacting with silicate melt (Fig. 12A). The high R factors (> 1,000) achieved would have largely diluted the geochemical (S/Se)–isotopic (Δ^33^S) signatures of the assimilated Archean S, such that most of the sulfide liquid would have been characterized by mantle-like signatures, apart from those that interacted with lower volumes of silicate melt.

During the low-energy stage of conduit development when the magmatic system was beginning to wane, the thinner morphology and/or shallow emplacement of the Current–Bridge and Beaver–Cloud zones promoted their early and rapid crystallization, closing them off to further magmatic activity (Fig. 12B). These regions of the conduit are characterized by high R factor, Pd-rich sulfides. The SEA was still magmatically active during this stage, allowing the composition of the resident magma and sulfide liquid to be further modified by magmatic processes. Restriction of magma flow to the portion of the conduit hosted by metasedimentary country rock, and its eventual stagnation in this area, allowed local S to be added to the magma, generating additional sulfide liquid with distinct S isotope compositions. During periods of magmatic quiescence, olivine, clinopyroxene, and sulfide liquid drained down the conduit and, potentially, accumulated in the feeder zone, decreasing the sulfide abundance and Pd content of the SEA (Fig. 12B). The eventual cessation of the magmatic system allowed the magma in the SEA to evolve via essentially closed-system fractional crystallization, generating the systematic change in lithology from primitive to more evolved rocks up stratigraphy (Fig. 12B). After crystallization, circulation of hydrothermal fluids altered the host rocks and mineralization, which remobilized some of the metals.

### Comparison to the architecture of other Ni–Cu–PGE deposits

Intrusions can exhibit a range of geometries, including channelized subvolcanic sills (e.g., Norilsk, Russia and Uitkomst, South Africa; Gauert et al. 1995; Naldrett and Lightfoot 1999; Maier et al. 2004), tube-like conduits (e.g., Nebo Babel, Australia and Limoiera, Brazil; Seat et al. 2007; Mota-e-Silva et al. 2013), and feeder dikes that link vertically separated sills (e.g., Voisey’s Bay, Canada; Evans-Lamswood et al. 2000). Although the Current deposit is classified as a conduit-type intrusion, its architecture falls into multiple of the aforementioned intrusion geometries depending on the location within the mineralized system. Specifically, the granite-hosted Current–Bridge Zone is characterized by a tube-like geometry similar to Nebo Babel, Limoiera, and Uitkomst, whereas the sedimentary-hosted Beaver–Cloud and 437–SEA zones have more ribbon-like geometries, being notably wider than they are thick, similar to Norilsk (Figs. 2 and 3). This change in geometry occurs abruptly at the contact between the rheologically distinct granite and sedimentary country rocks (Figs. 2 and 3). Although it is known that crustal-scale weaknesses exhibit fundamental controls on magma migration and intrusion geometry, the Current intrusion is one of few magmatic systems in which this is unambiguously demonstrated. This contrasts with Nebo Babel, Limoiera, and Uitkomst, which intruded into either intrusive or sedimentary country rock and exhibit comparatively invariable geometries along their lengths (Maier et al. 2004; Seat et al. 2007; Mota-e-Silva et al. 2013). One peculiarity of the geometry of the Current intrusion is that, unlike most magmatic intrusions, it thickens towards the feeder zone (Fig. 2). Considering that both the Beaver–Cloud Zone and SEA coincide with the Quetico and Escape Lake fault zones, respectively, and both zones are thick relative to the Current–Bridge Zone (Fig. 2), it is suggested that this thickening resulted from exploitation of a crustal weakness. The Current intrusion, therefore, serves as a prime example of how country rock rheology and crustal weakness control intrusion architecture.

## Conclusions

The Current deposit represents one of the best examples of a conduit-type Ni–Cu–PGE sulfide deposit. This tube-shaped intrusion was emplaced into Archean metasedimentary and granitic rocks, and records a variety of magmatic and post-magmatic processes in the texture, and trace-element and S isotope composition of its diverse BMS assemblage.

1. Saturation of the Current magmas in sulfide and generation of a sulfide liquid resulted from addition of external S from at least two sources, one located at depth (Δ^33^S < 3‰) and the other being the local Archean metasedimentary country rocks (Δ^33^S < 0.3‰).
2. The Current intrusion crystallized sequentially, from the Current–Bridge Zone towards the 437–SEA Zone. This, along with the elevated Cu/Pd ratios, distinct source of S, and igneous layering in the 437–SEA Zone, suggests that it represents the feeder channel to the Current deposit.
3. The BMS mineralogy was modified by the circulation of hydrothermal fluids through the rocks, with pyrrhotite being replaced by pyrite and pentlandite being replaced by millerite. This fluid activity mobilized several metals and semi-metals, including Fe, Ni, S, Se, Co, Cu, As, and Ag, but did not affect the PGE.

### Supplementary Information

Below is the link to the electronic supplementary material.

Supplementary file1 (DOCX 3303 KB)

Supplementary file2 (XLSX 426 KB)
