# Supplementary material for: Characterizing the supra- and subsolidus processes that generated the Current PGE–Cu–Ni deposit, Thunder Bay North Intrusive Complex, Canada: insights from trace elements and multiple S isotopes of sulfides
Source: Miner Depos. 2023 Jul 29;58(8):1559–81. doi: 10.1007/s00126-023-01193-9 (PMC10598094; doi:10.1007/s00126-023-01193-9)
Supplement: Supplementary file 1 — Supplementary file1 (DOCX 3303 KB) [file 126_2023_1193_MOESM1_ESM.docx]

Characterizing the supra- and subsolidus processes that generated the Current PGE–Cu–Ni deposit, Thunder Bay North Intrusive Complex, Canada: insights from trace elements and multiple S isotopes of sulfides

Brzozowski, M.^1,2*^, Hollings, P.^2^, Heggie, G.^3^, MacTavish, A.^3^, Wilton, D.^4^, Evans-Lamswood, D.^3^

*^1^British Columbia Geological Survey, 1810 Blanshard Street, Victoria, BC, V8T 4J1, Canada*

*^2^Department of Geology, Lakehead University, 955 Oliver Road, Thunder Bay, ON, P7B 5E1, Canada*

*^3^Clean Air Metals, 1004 Alloy Drive, Thunder Bay, ON, P7B 6A5, Canada*

*^4^Earth Sciences, Memorial University, 230 Elizabeth Avenue, St. John’s, NL, A1C 5S7, Canada*

*Corresponding author: Matthew Brzozowski (matt.brzozow@gmail.com)

Electronic Supplementary Material 1

Mineral Liberation Analysis

The SEM utilized for Mineral Liberation Analysis was operated at an accelerating voltage of 20 kV and beam current of 10 nA. Mineral liberation analyses were conducted using a frame size of 1.5 x 1.5 mm at 500 pixels per frame, a step size of 10 pixels, and an X-ray acquisition time of 12 ms. Minerals were identified by comparing observed EDX spectra to the spectra of known minerals housed in the species identification protocol developed by Memorial University of Newfoundland (e.g., Wilton et al. 2017).

Bulk-rock geochemistry

Bulk-rock Pd and Pt concentrations were determined by aqua regia digestion with atomic absorption spectroscopy finish, or fire assay with either atomic absorption or inductively coupled plasma mass spectrometry (ICP–MS) finishes; overlimits in the latter were re-analyzed by ICP–AES. Iridium was determined by fire assay with ICP–MS finish. Copper and S were determined by aqua regia digestion with atomic absorption spectroscopy finish or by four acid digestion with ICP–AES finish; the S content of a limited number of samples (0.4% of the database) was determined by LECO. Selenium was determined by ICP–MS following either four acid or aqua regia digestion.

LA–ICP–MS analysis of base-metal sulfides

The laser was operated at an energy of 3 mJ, a repetition rate of 20 Hz, and a spot size of 20 µm. Analyses represent traverses across BMS grains at a traverse speed of 5 µm/s and included 30 s of gas background collection followed by up to 90 s of ablation and signal collection. The masses determined were ^29^Si, ^33^S, ^34^S, ^51^V, ^57^Fe, ^59^Co, ^61^Ni, ^63^Cu, ^65^Cu, ^66^Zn, ^68^Zn, ^75^As, ^77^Se, ^78^Se, ^82^Se, ^101^Ru, ^103^Rh, ^105^Pd, ^106^Pd, ^108^Pd, ^109^Ag, ^111^Cd, ^118^Sn, ^120^Sn, ^121^Sb, ^123^Sb, ^125^Te, ^185^Re, ^189^Os, ^193^Ir, ^194^Pt, ^197^Au, ^208^Pb, and ^209^Bi. Data reduction was carried out using Iolite 4 (Paton et al. 2011). Iron was used as the internal standard for all analyses; it was determined using a Hitachi Su-70 Schottky Field Emission SEM–EDS at the Lakehead University Instrumentation Laboratory operated at an accelerating voltage of 20 kV and counting times of 60 s. Instrument calibration was achieved using ZnS and chalcopyrite standards produced by Micro-Analysis Consultants Ltd., and a NiS standard produced by the Carnegie Science Geophysical Laboratory. MASS-1 was used as the calibration standard for trace elements (excluding PGE and Au) (Wilson et al. 2002). The Au, Ir, Os, Pd, Pt, Rh, and Ru contents of BMS were calibrated using Po725 (Sylvester et al. 2005). The concentrations of Pb and Te were calibrated using published values in MASS-1 (76.5 ppm and 15.1 ppm, respectively; Feng et al. 2018; Lorand et al. 2018). Copper and Zn argide interferences on Rh and Pd (^63^Cu^40^Ar on ^103^Rh, ^65^Cu^40^Ar on ^105^Pd, ^66^Zn^40^Ar on ^106^Pd, and ^68^Zn^40^Ar on ^108^Pd) were corrected for by analyzing pure Cu and Zn metals at the beginning of each analytical session, and applying a correction factor to the raw counts, as described by Brzozowski et al. (2020). Interferences of ^106^Cd and ^108^Cd on ^106^Pd and ^108^Pd, respectively, were corrected by applying a correction factor to the calculated concentrations (^106^Pd = 0.043* Cd ppm and ^108^Pd = 0.034 * Cd ppm).

Typical laser ablation (LA) spectra of chalcopyrite, pyrrhotite, pentlandite, millerite, and pyrite are illustrated in Figure S1. As the goals of the study require characterizing the composition of BMS, inclusions of silicates and oxides (monitored using Si and V, respectively), BMS (e.g., sphalerite and pyrrhotite inclusions in chalcopyrite), and platinum-group minerals (PGM) were excluded from the integration regions when possible. Sphalerite inclusions, which are most common in chalcopyrite and millerite, were identified as coincident spikes of Zn ± Co ± Cd in LA spectra (Fig. S1a, d), whereas inclusions of pyrrhotite–pentlandite and chalcopyrite occur as coincident peaks of Co ± Ni and Cu ± Zn, respectively (Fig. S1b). Platinum-group minerals were identified based on the coincidence of PGE (mostly Pt, Pd, and Ir) with post-transition metals (mostly Bi and Pb) (Fig. S1a, b, c, e). Platinum-group elements generally do not occur in the structures of chalcopyrite, pyrrhotite, and millerite, however, Pd–Rh–Ir–Os and Pd–Pt occur in the structures of pentlandite and pyrite, respectively (Fig. S1c, d). Apart from pyrite, the BMS do not exhibit any zonation or heterogeneity in their major–trace-element chemistry (apart from mineral inclusions). Pyrite, however, is characterized by variable Ni–Co ± As concentrations, with regions of high and low Ni–Co ± As (Fig. S1e). This variation can sometimes correlate with the PGE content of the pyrite, with the high Ni–Co ± As regions containing higher PGE contents (Fig. S1e); these regions were integrated separately. It is important to note that the zonation in pyrite or the occurrence of BMS inclusions generally does not affect the Se content of the host BMS (Fig. S1).

Sulfur isotope analyses of base-metal sulfides

Resin mounts containing polished samples were trimmed to accommodate a standard block containing matrix-match standards that were placed within the SIMS sample holder. The mounts were coated with a ~20 nm layer of gold to prevent charging. The sample surface was sputtered over a 10 x 10 µm area with a 10 kV, Gaussian Cs+ beam operated at an intensity of ~2.5 nA and total impact energy of 20 keV. Sulfur isotopes 32S, 33S, and 34S were collected simultaneously using Faraday cup detectors fitted with 1010 Ω (L’2, 32S) and 1011 Ω (L1, 33S and H1, 34S) resistors, whereas 36S was collected using an electron multiplier (H2), all of which were operated at a mass resolution of ~2500. The 32S1H and 33S peaks are not completely resolved under these conditions, so the magnetic field was offset slightly to the low-mass side to avoid interference from 32S1H on the 33S peak. Each analysis included 30 s of pre-sputtering over a 15 x 15 µm area, followed by automatic centering of the secondary ions in the field aperture, and then 30 four-second cycles of data acquisition. Each S isotope composition represents the average of up to 15 spot analyses within a BMS grain (or nearby grain where sulfides are volumetrically small), with internal precision represented as two standard deviations from this average. Instrumental drift was monitored by analyzing two standards every 9 (typically 5–6) sample analyses. Instrumental mass fractionation was corrected using the matrix-matched reference material for pyrrhotite (Alexo), pyrite (Sierra), chalcopyrite (Nifty-b), and pentlandite (VMSO) (LaFlamme et al. 2016). Data processing follows the procedure described in Laflamme et al. (2016).


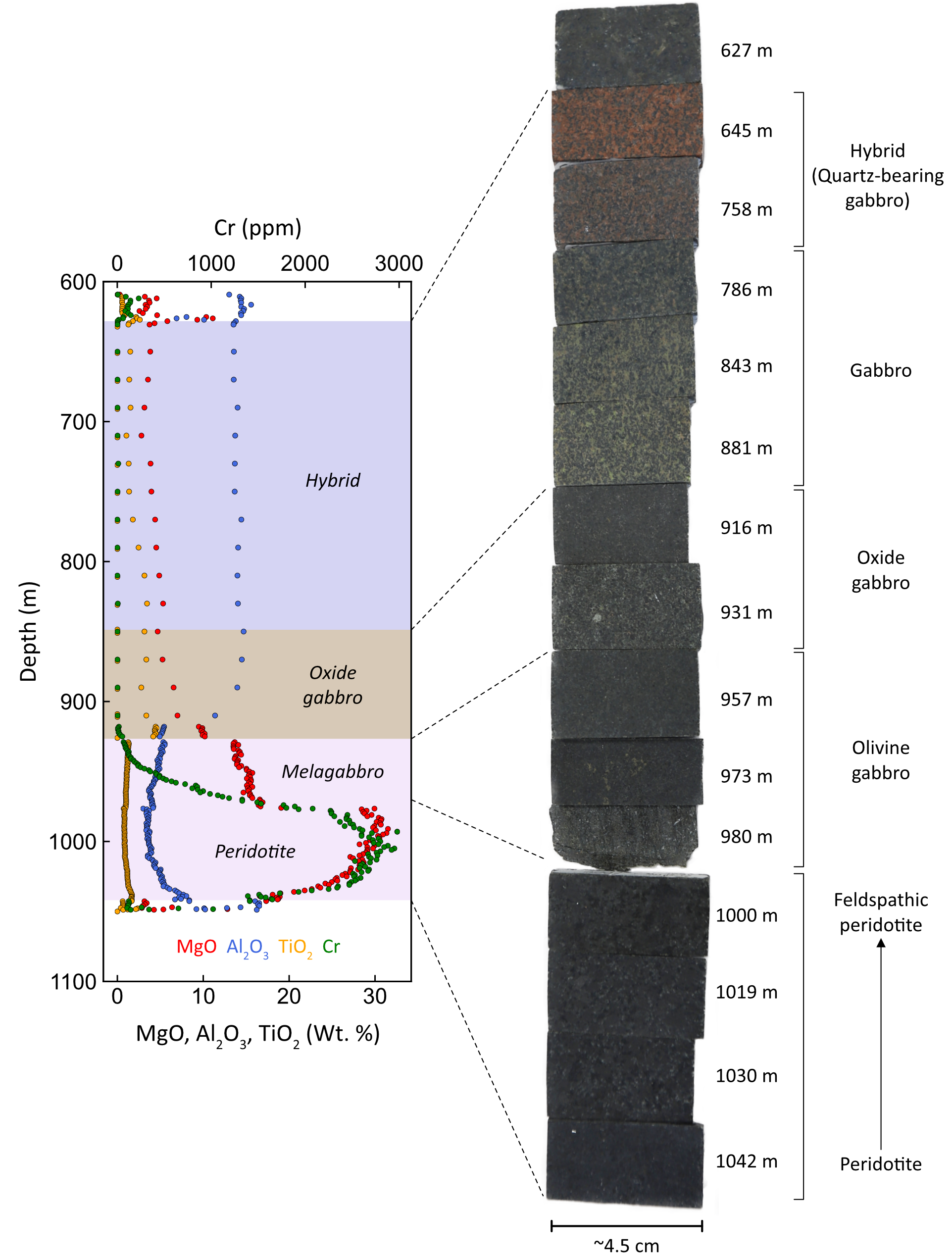


**Figure S1** Variations in bulk-rock MgO, Al_2_O_3_, TiO_2_, and Cr along drill hole SEA08-02, which intersects the Southeast Anomaly. Next to the downhole plot are a representative set of samples of lithologies along the length of drillhole SEA08-02.


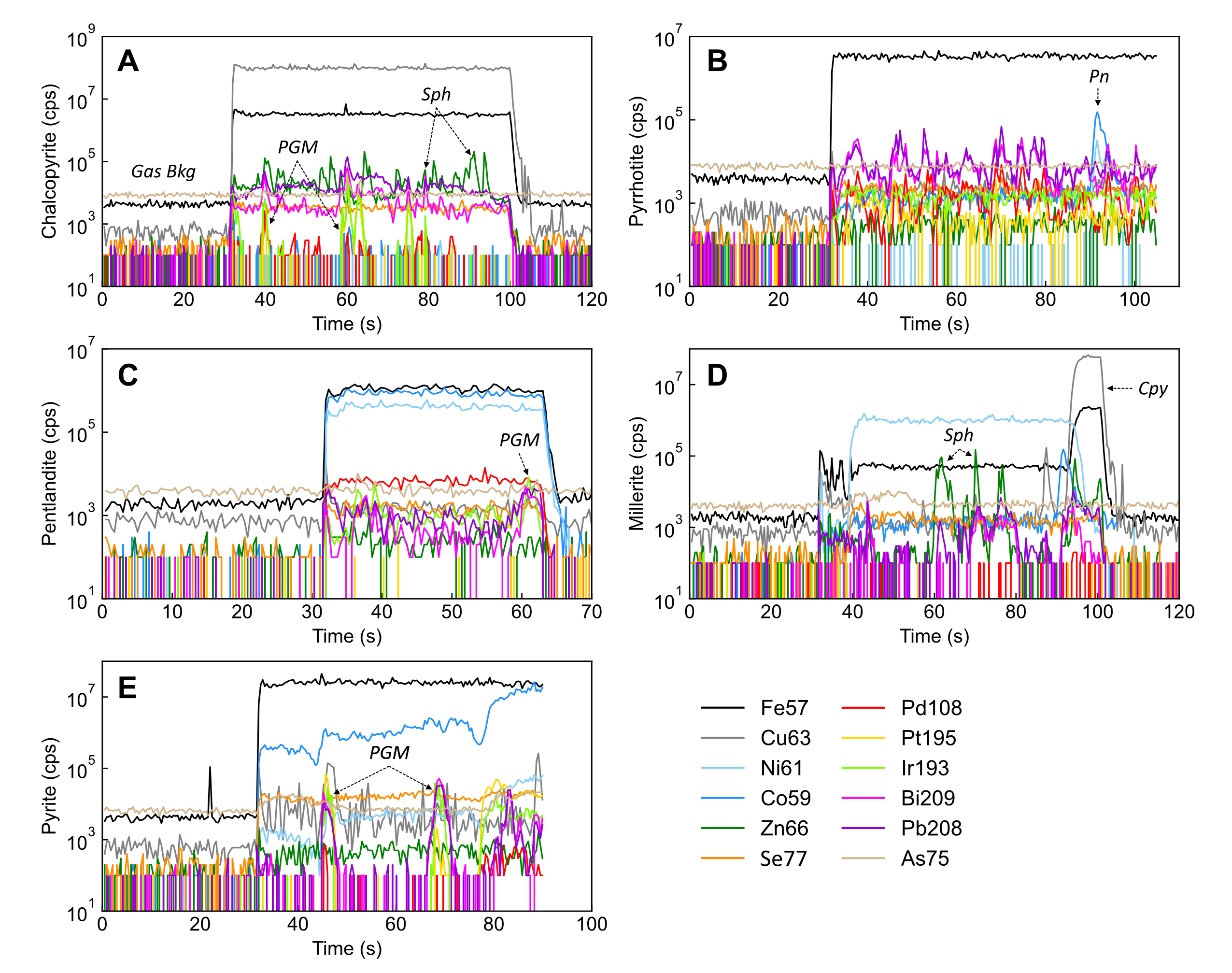


**Figure S2** Laser ablation spectra illustrating the variation in counts per second for base- and precious metals, and semi-metals in (A) chalcopyrite, (B) pyrrhotite, (C) pentlandite, (D) millerite, and (E) pyrite.


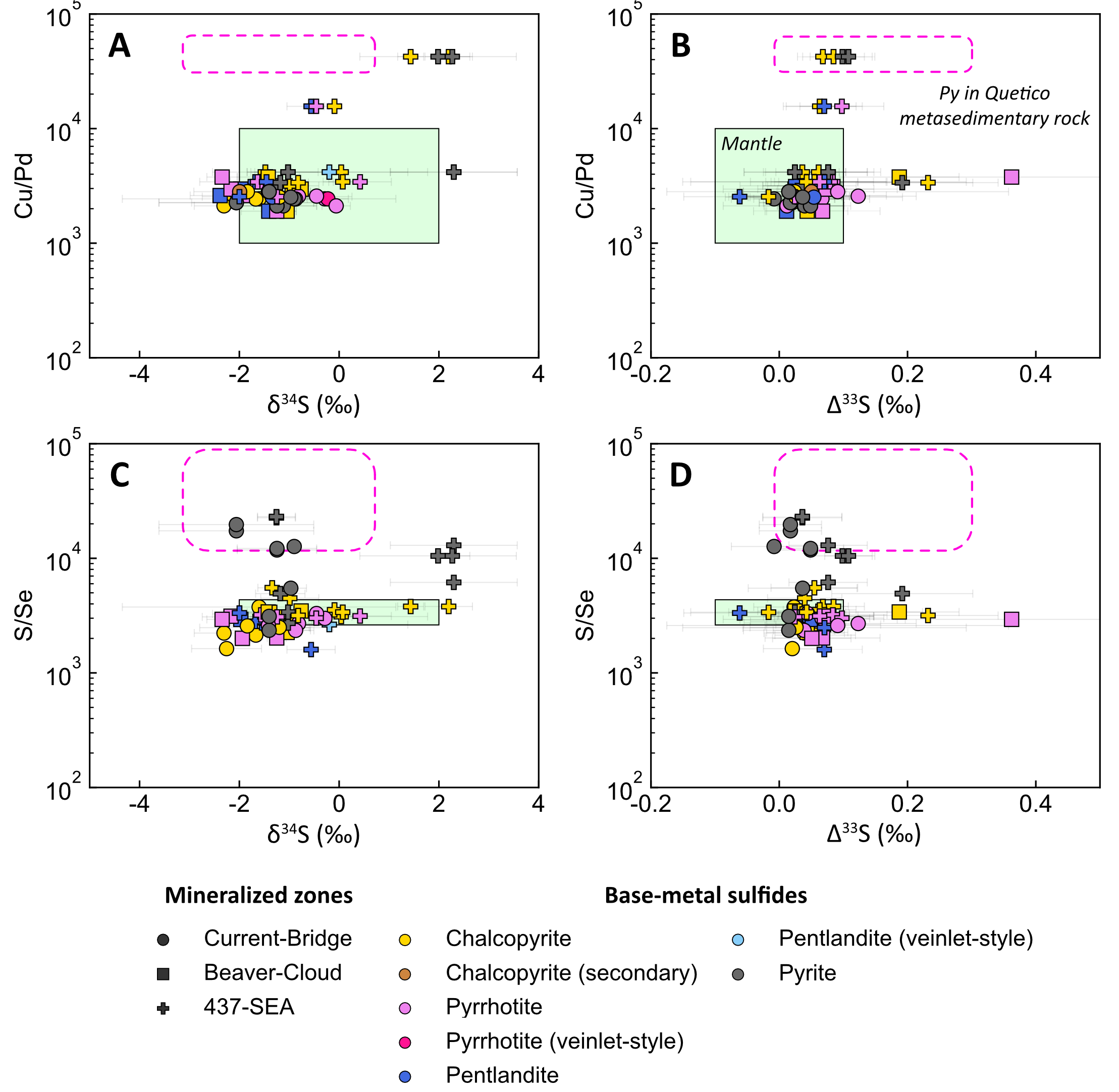


**Figure S3** Binary diagrams illustrating the variation in (A) δ^34^S–bulk-rock Cu/Pd, (B) Δ^33^S–bulk-rock Cu/Pd, (C) δ^34^S–S/Se, and (D) Δ^33^S–S/Se of base-metal sulfides. The mantle range for Δ^33^S is from Farquhar (2002) and Bekker et al. (2009), and for δ^34^S is from Lesher and Burnham (2001) and Ripley and Li (2003). The mantle ranges for Cu/Pd (1,000–10,000) and S/Se (2,632–4,350) are the same as in Figure 8. The pink, dashed field highlights the composition of pyrite from Quetico metasedimentary rocks from Caglioti (2023). Error bars for S isotopes are 2σ.


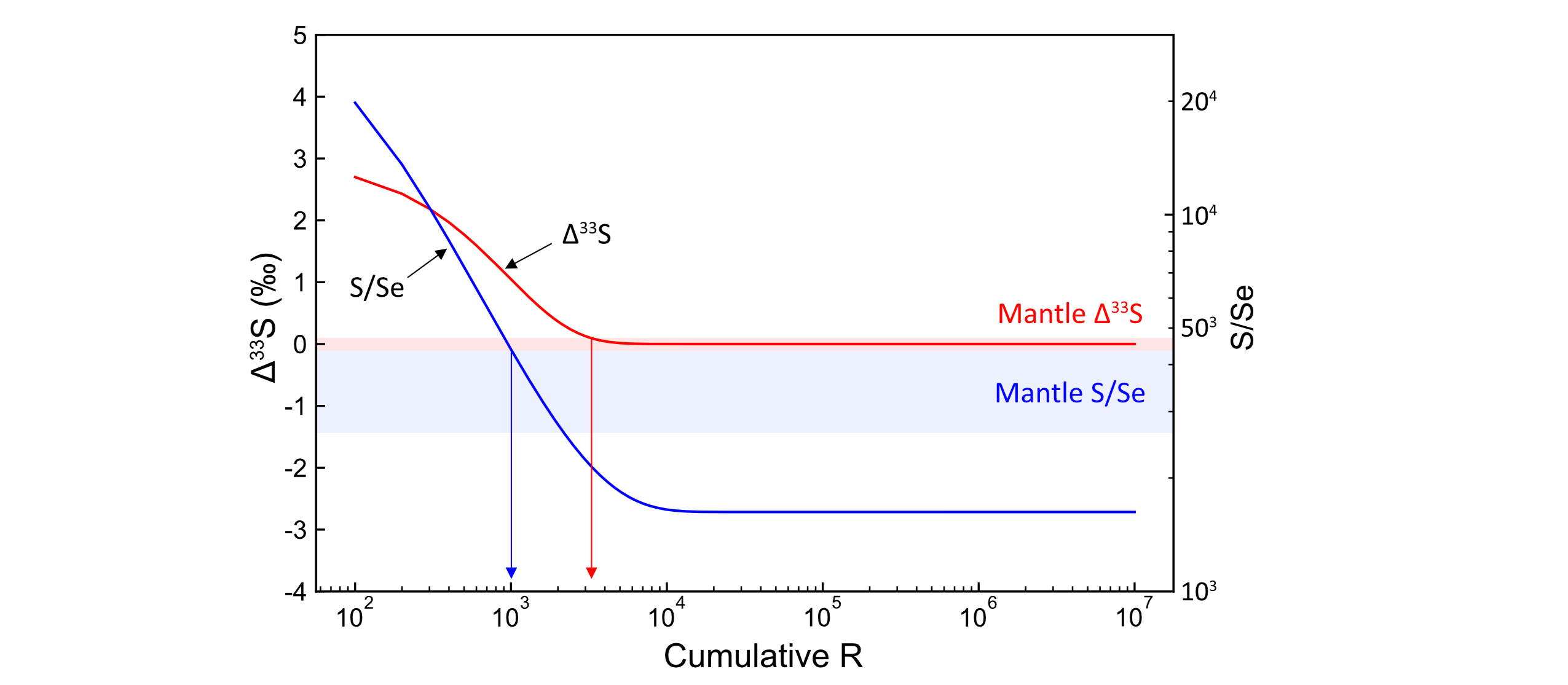


**Figure S4** Binary diagram illustrating the modeled variation in Δ^33^S and S/Se as a function of variations in cumulative R factor. Note that Δ^33^S and S/Se are on separate y axes and use different scales. The mantle range for Δ^33^S is from Farquhar (2002) and Bekker et al. (2009), and for S/Se is from Eckstrand and Hulbert (1987) and Palme and O’Neil (2014).
